# Microbiome Modulation with *Lactobacillus rhamnosus* GG Potentiates Curcumin's Efficacy in Reversing Gemcitabine Resistance of Gallbladder Cancer through Gut Microbiota-PI3K/AKT Axis

**DOI:** 10.4014/jmb.2601.01007

**Published:** 2026-04-21

**Authors:** Yanliang Li, Siqiang Niu, Haipeng Wang, Jianhua Li

**Affiliations:** 1 Department of General Surgery, Xinxiang City Central Hospital, Xinxiang 453000, Henan, P. R. China; 2 Department of Critical Care Medicine, Xinxiang 453000, Henan, P. R. China

**Keywords:** Curcumin, Probiotics, Gallbladder cancer, Chemotherapy resistance, Gut microbiota, PI3K/AKT pathway

## Abstract

Gemcitabine (GEM) resistance remains a major challenge in the treatment of gallbladder cancer (GBC). This study investigated the synergistic effect of curcumin (CUR) combined with the probiotic *Lactobacillus rhamnosus* GG (LGG) in reversing chemoresistance through modulation of the gut microbiota. In GEM-resistant GBC-SD cells, the CUR–LGG combination significantly inhibited cell proliferation, suppressed migration and invasion, and induced apoptosis, as demonstrated by CCK-8, wound healing, Transwell, and flow cytometry assays. Western blot analysis revealed corresponding regulation of proliferation markers (Ki67, PCNA), apoptosis-related proteins (Bcl-2, Bax, cleaved Caspase-3), and epithelial–mesenchymal transition markers. In xenograft models, the combined treatment markedly suppressed tumor growth and altered gut microbial composition, increasing beneficial bacteria (*Lactobacillus*, *Bifidobacterium*) while reducing pathogenic taxa. LC–MS analysis further demonstrated restoration of bile acid homeostasis, characterized by elevated primary bile acids (GCA, CDCA) and decreased secondary bile acids (DCA, LCA). Mechanistically, the intervention significantly inhibited PI3K/AKT signaling, as confirmed by Western blot and immunohistochemistry. Bioinformatic analysis further identified PI3K/AKT as a central regulatory pathway. These findings indicate that probiotic-assisted CUR therapy reverses GEM resistance by remodeling the gut microbiota and its metabolic outputs, thereby suppressing oncogenic signaling pathways. This strategy provides a promising microbiota-based approach for improving therapeutic outcomes in GBC.

## Introduction

Gallbladder cancer (GBC) is a highly aggressive malignancy of the digestive system, and its incidence has shown an increasing trend worldwide [1, 2]. Due to early-stage symptoms that are often subtle and nonspecific, most patients are diagnosed at advanced stages, resulting in low surgical resection rates and poor prognosis [3, 4]. Currently, gemcitabine (GEM) combined with cisplatin remains the standard first-line chemotherapy for advanced GBC. Despite its initial therapeutic benefit, clinical studies have reported that resistance frequently emerges during treatment, which contributes to rapid disease progression and limited overall survival [5, 6]. Recent studies suggest that combination strategies integrating natural bioactive compounds with microbiota remodeling may provide novel therapeutic opportunities in cancer treatment and potentially overcome chemotherapy resistance [7-9]. Therefore, elucidating the molecular mechanisms underlying drug resistance in GBC and developing multi-targeted therapeutic strategies are of considerable clinical significance.

Curcumin (CUR), a naturally occurring polyphenol derived from *Curcuma longa*, exhibits anti-inflammatory, antioxidant, and anticancer activities and has been widely investigated as an adjunct therapy in multiple cancers [10, 11]. Evidence indicates that CUR suppresses tumor cell proliferation, migration, and invasion primarily by inhibiting pathways such as NF-κB and STAT3, while also enhancing the cytotoxic efficacy of conventional chemotherapeutic agents [12, 13]. In GBC, aberrant activation of the PI3K/AKT axis represents a key molecular feature driving tumor initiation and malignant progression [14, 15]. Activation of this pathway has also been closely linked to the development of chemoresistance in tumor cells [16, 17]. Recent studies have demonstrated that inhibition of PI3K/AKT signaling significantly enhances the sensitivity of GBC cells to GEM, highlighting its central role in regulating chemosensitivity [18]. However, the clinical use of CUR is restricted by its poor aqueous solubility and low bioavailability, emphasizing the need for rational combination strategies to improve its therapeutic efficacy [19, 20]. Growing evidence suggests that the gut microbiota plays a critical role in tumor initiation, progression, and therapeutic responsiveness. Among probiotic strains, *Lactobacillus rhamnosus* GG (LGG) has been reported to restore intestinal microbial homeostasis, enhance host immune responses, and regulate bile acid metabolism, thereby contributing to a more favorable tumor microenvironment (TME) [21, 22]. Moreover, LGG may indirectly modulate oncogenic signaling pathways, including PI3K/AKT and Wnt/β-catenin, through alterations in short-chain fatty acid (SCFA) production and bile acid transformation, which ultimately improves tumor chemosensitivity [23-25]. Therefore, CUR combined with LGG may exert cooperative antitumor activity by modulating the microbiota–metabolism–immunity axis. Nevertheless, the molecular basis of their synergistic effects in overcoming GBC chemoresistance has not yet been fully elucidated.

The development of GEM resistance in GBC is driven by multifaceted molecular alterations, with aberrant activation of the PI3K/AKT signaling cascade identified as a principal contributor [26, 27]. This pathway governs diverse cellular functions, including proliferation, survival, migration, and metabolic regulation. Persistent PI3K/AKT activation inhibits apoptosis, promotes epithelial–mesenchymal transition (EMT), and upregulates drug efflux transporters such as P-glycoprotein (P-gp) and breast cancer resistance protein (BCRP), thereby decreasing intracellular accumulation of chemotherapeutic drugs [28, 29]. Increasing evidence further suggests that gut microbiota dysbiosis can activate PI3K/AKT signaling through alterations in bile acid metabolism. In particular, the accumulation of secondary bile acids such as deoxycholic acid (DCA) and lithocholic acid (LCA) has been reported to promote tumor progression and drug resistance [30, 31]. However, in GBC, the regulatory role of the gut microbiota-bile acid-PI3K/AKT axis has not been systematically studied, particularly lacking experimental evidence on whether the combination of CUR and LGG can affect GEM resistance through this pathway [32]. Therefore, elucidating the interactions among gut microbiota remodeling, bile acid metabolism, and PI3K/AKT signaling may provide new insights into the mechanisms underlying chemoresistance in GBC and support the development of microecology-based therapeutic strategies.

This study was designed to comprehensively assess the synergistic effect of CUR and LGG in overcoming GEM resistance in GBC, and to elucidate the underlying regulatory mechanisms mediated through the “gut microbiota–bile acid–PI3K/AKT” axis. GEM-resistant GBC-SD cells were established for *in vitro* experiments, and assays including Cell Counting Kit-8 (CCK-8), 5-ethynyl-2?-deoxyuridine (EdU) staining, and flow cytometry were used to assess cell proliferation, apoptosis, and migration following CUR and LGG treatment. *In vivo*, xenograft nude mouse models were constructed to further investigate therapeutic efficacy. Gut microbiota composition was analyzed using quantitative polymerase chain reaction (qPCR), bile acid metabolic profiles were determined by liquid chromatography–mass spectrometry (LC–MS), and the expression of key PI3K/AKT signaling molecules was examined by Western blot (WB) analysis. Overall, the present study seeks to elucidate the synergistic mechanism by which CUR combined with LGG reverses GBC chemoresistance. The findings are expected to provide new evidence supporting natural product–probiotic combination therapy, clarify the relationship between gut microbiota remodeling, bile acid metabolism, and PI3K/AKT signaling, and establish a theoretical basis for developing low-toxicity and high-efficacy anticancer strategies based on microecological regulation. These insights may contribute to personalized therapeutic approaches for GBC and advance precision medicine strategies targeting TME and metabolic regulation.

## Materials and Methods

### Ethical Statement

All animal procedures complied with institutional and national guidelines for the care and use of laboratory animals. The experimental protocol was reviewed and approved by the Institutional Animal Care and Use Committee (IACUC). Animals were housed under standardized conditions following the principles of the 3Rs (Replacement, Reduction, and Refinement), and all procedures were performed to minimize pain and distress. At the end of the study, mice were euthanized humanely under diethyl ether anesthesia in compliance with AVMA guidelines.

### Ultrasound-Assisted Extraction (UAE) of CUR

Fresh rhizomes of *Curcuma longa* were thoroughly washed, oven-dried at 60°C for 48 h (UN30, Memmert, Germany), and ground into fine powder. Fifty grams of the powder was mixed with 500 mL of absolute ethanol (≥99.5%, 459844, Sigma-Aldrich, USA) and subjected to UAE using an ultrasonic processor (Branson Ultrasonics, USA) at 400 W and 40 kHz for 60 min, with the extraction temperature maintained at 30°C. After filtration, the solution was evaporated under reduced pressure at 40°C and 100 mbar using a rotary evaporator (R-300, Büchi, Switzerland). The crude extract was then collected and preserved at -20°C until further analysis.

### CUR Purification

The crude extract was further purified using a Waters Alliance high-performance liquid chromatography (HPLC) system (Waters, USA) equipped with an XBridge C18 column (5 μm, 4.6 × 250 mm; 186003117, Waters). The mobile phases consisted of 2% acetic acid in water (A) and 2% acetic acid in acetonitrile (B). The gradient elution program was as follows: 0–3 min, 10% B; 8 min, 20% B; 13 min, 25% B; 18 min, 35% B; 28–33 min, 55% B (held for 3 min), followed by re-equilibration to the initial conditions. The flow rate was maintained at 1.0 mL/min, and detection was performed at 420 nm. The major peak fraction was collected and further identified by mass spectrometry. CUR purity was confirmed to be ≥95%. Purified CUR was dissolved in DMSO (276855, Sigma-Aldrich) to obtain a 10 mg/mL stock solution, which was stored at -20°C in the dark until further use.

### Active Component Analysis

An aliquot of the crude extract was dissolved in methanol (A452-4, Thermo Fisher Scientific, USA) to a concentration of 1 mg/mL and passed through a 0.22 μm organic membrane filter (Z290807, Sigma-Aldrich). The filtrate was subsequently analyzed using an LC–MS system (Agilent Technologies, USA). Chromatographic separation was achieved using an Agilent Eclipse Plus C18 column (2.1 × 150 mm, 1.8 μm; 959759-902, Agilent Technologies). The mobile phase consisted of ultrapure water with 0.1% formic acid (A) and acetonitrile with 0.1% formic acid (B). Gradient elution was performed as follows: 0–5 min, 50–85% B; 5–7 min, 85% B; 7–8 min, 85–50% B; and 8–10 min, 50% B. Mass spectrometric detection was carried out in negative ion mode using nitrogen (≥99.999%) as the carrier gas. The nebulizer gas flow was set at 5 L/min, while the sheath gas flow rate and temperature were maintained at 11 L/min and 250 °C, respectively. The ion source temperature was 300°C, and the capillary and nozzle voltages were adjusted to 3500 V and 500 V. Multiple reaction monitoring (MRM) mode was used to detect CUR and its analogs demethoxycurcumin (DMC) and bisdemethoxycurcumin (BDMC). The monitored ion transitions were m/z 367.1 → 217.0 for CUR, 337.1 → 217.0 for DMC, and 307.1 → 217.0 for BDMC. Collision energies were set at 4 V and 12 V. Quantification was performed using calibration curves. Extracted ion chromatograms were used for semi-quantitative analysis based on peak area normalization, and the relative percentages of each compound in the crude and purified extracts were calculated [33, 34].

### Preparation of *Lactobacillus rhamnosus* GG Cell-Free Supernatant

LGG (Catalog No.: 01090P, Microbiologics, USA) was inoculated on MRS agar medium (HB0384-51, Hopebio, China) and cultured

anaerobically at 37°C for 24 h. A single colony was inoculated into MRS broth (HB0384-1, Hopebio) and cultured anaerobically at 37°C for 24 h. The culture was then centrifuged (8,000 × g, 4°C, 10 min) to remove cells, and the supernatant was sterilized through a 0.22 μm filter and stored at -80°C until use.

### Cell Culture and Treatment

Human GBC-SD cells (CBP60716, Cobioer, China) were cultured in RPMI-1640 medium (12633012, Gibco, USA) supplemented with 10% fetal bovine serum (FBS; 10099141C, Gibco) and 1% penicillin-streptomycin (V900929, Sigma) and maintained at 37°C in a humidified incubator with 5% CO_2_.

To establish GEM (S1714, Selleck, USA)-resistant cell lines, parental GBC-SD cells were initially exposed to 1 μM GEM. The drug concentration was gradually increased (1–12 μM) over approximately 20 passages until stable drug tolerance was achieved. Control cells were passaged under identical conditions with an equivalent volume of DMSO as the solvent control. The resulting resistant subline exhibited stable tolerance to 5 μM GEM. To verify the resistant phenotype, parental and resistant cells were subjected to GEM sensitivity testing using the CCK-8 assay. Cells were seeded in 96-well plates and exposed to GEM at 0, 0.1, 1, 5, 10, or 20 μM for 48 h. Cell viability and IC_50_ values were then determined. The resistance index (RI) was calculated as the ratio of IC_50_ (resistant line) to IC_50_ (parental line) [35].

The study included five treatment groups: PBS group (equal volume PBS treatment as solvent control), CUR group (40 μg/mL CUR treatment), GEM group (5 μM GEM treatment), CUR+LGG group (40 μg/mL CUR + 25% v/v LGG supernatant), and GEM+CUR+LGG group (5 μM GEM + 40 μg/mL CUR + 25% v/v LGG supernatant).

### CCK-8 Assay

Cells were seeded in 96-well plates and treated with the indicated reagents for 12, 24, 36, or 48 h. Then, 10 μL of CCK-8 reagent (C0038, Beyotime, China) mixed with 100 μL of culture medium was added to each well and incubated for 45 min. Absorbance (OD) was measured at 450 nm using a microplate reader (BioTek, USA) to determine cell viability.

### EdU Proliferation Assay

Cells were cultured in 96-well plates and processed for EdU detection (C0081S, Beyotime). Nuclei were counterstained with DAPI (C1006, Beyotime), and proliferating cells were visualized under a fluorescence microscope.

### Colony Formation Assay

Treated cells were seeded in 6-well plates at 500 cells per well and cultured for 10 days to allow colony formation. Colonies were fixed with 4% paraformaldehyde (PFA; P0099, Beyotime) and stained with 0.1% crystal violet (C0121, Beyotime). Images were acquired under a microscope (Leica, Germany), and colony numbers were quantified in randomly selected fields.

### Cell Apoptosis Detection

Cell apoptosis was analyzed using the Annexin V-FITC/PI Apoptosis Detection Kit (11684795910, Roche, Switzerland). After 48 h of treatment, cells were collected, washed twice with cold PBS, and centrifuged at 2500 rpm for 5 min. The cell pellet was resuspended in 100 μL of cold 1× binding buffer containing 1 μL Annexin V-FITC and 5 μL propidium iodide (PI), followed by incubation in the dark for 15 min. Subsequently, 400 μL of cold 1× binding buffer was added, and samples were gently mixed. Apoptotic cells were analyzed within 30 min using a flow cytometer (DxFLEX, Beckman Coulter, USA).

### Wound Healing Assay

Cells were seeded in 6-well plates and cultured for 24 h to form a confluent monolayer. A linear scratch was made with a sterile pipette tip, and debris was removed by PBS washing. The medium was replaced with serum-free medium, and images were captured at 0 and 24 hours. Migration distances were measured using ImageJ (version 1.53a, NIH, USA).

### Transwell Invasion Assay

Cells suspended in serum-free medium were seeded into Matrigel-coated Transwell inserts (3422, Corning, USA; 356234, BD Biosciences, USA). The lower chamber contained RPMI-1640 medium with 10% FBS as a chemoattractant. After 24 h, invaded cells were fixed with 4% PFA, stained with 0.5% crystal violet for 15 min, rinsed with PBS, and imaged under an optical microscope. Cell numbers were quantified in randomly selected fields.

### WB Analysis

Total proteins were extracted from cells and tissues using RIPA lysis buffer (P0013B, Beyotime) containing 1% PMSF. Protein concentrations were determined with a BCA assay kit (P0011, Beyotime) and adjusted to 1 μg/μL. Samples were denatured by boiling at 100°C for 10 min and stored at -80°C until use. Proteins (50 μg per lane) were separated by SDS–PAGE (8–12% gels, depending on target molecular weight) and electrophoresed at 80 V for 30 min, then 120 V for 90 min, followed by transfer onto PVDF membranes (1620177, Bio-Rad, USA) at 250 mA for 90 min.

After transfer, membranes were blocked with 5% skim milk prepared in Tris-buffered saline containing 0.1% Tween-20 (TBST) for 1 h at room temperature. Membranes were then incubated with primary antibodies (listed in Table S1) overnight at 4°C, followed by three washes with TBST (10 min each). Subsequently, membranes were incubated with horseradish peroxidase (HRP)-conjugated goat anti-rabbit IgG (ab6721, 1:5000, Abcam, UK) or goat anti-mouse IgG (ab205719, 1:5000, Abcam) for 1 hours at room temperature and washed three times with TBST. Protein bands were visualized using an Image Quant LAS 4000C system (GE Healthcare, USA). GAPDH served as a loading control, and target protein levels were quantified relative to GAPDH using ImageJ. Experiments were performed in triplicate.

### FXR Agonist/Antagonist Experiments

To verify the critical role of FXR in mediating the resistance-reversal effect, we performed *in vitro* modulation with the FXR agonist GW4064 (HY-50108, MCE, USA) and the antagonist guggulsterone (GS, HY-N0612, MCE, USA). GEM-resistant GBC-SD cells were divided into four experimental groups: PBS, CUR+LGG, CUR+LGG+GW4064 (10 μM), and CUR+LGG+GS (32 μM). Agonist/antagonist was added 1 hours prior to CUR+LGG; cells were co-treated for 48 hours before collection [36].

### Quantitative Real-Time PCR (qRT-PCR)

Total RNA was extracted from cultured cells or tumor tissues using TRIzol reagent (15596026, Invitrogen, USA). RNA concentration and purity were assessed by NanoDrop 2000 (Thermo Fisher Scientific, USA). Complementary DNA (cDNA) was synthesized from 1 μg of total RNA using the PrimeScript RT Reagent Kit (RR047A, Takara, Japan). qPCR was performed with TB Green Premix Ex Taq II (RR820A, Takara) on a QuantStudio 5 system (Applied Biosystems, USA). Quantitative PCR reactions were prepared in a final volume of 20 μL containing TB Green Premix (10 μL), forward and reverse primers (0.8 μL each, 10 μM), cDNA template (2 μL), and nuclease-free water (6.4 μL). Thermal cycling was performed with an initial denaturation at 95°C for 30 s, followed by 40 amplification cycles consisting of 95°C for 5 s and 60°C for 30 s. Gene expression levels were normalized to GAPDH and calculated using the 2^−ΔΔCt^ method [35]. Primer sequences are listed in Table S2.

### Establishment of GBC Xenograft Mouse Model

Female BALB/c nude mice (nu/nu, 8–10 weeks old, 18–20 g; Vital River Laboratory Animal Technology Co., Ltd., China) were maintained under specific pathogen-free (SPF) conditions (22 ± 2°C, 50–60% humidity, 12 hours light/dark cycle) with free access to autoclaved chow and purified water. To establish the xenograft model, logarithmically growing cells were digested with 0.25% trypsin (25200056, Sigma-Aldrich), centrifuged at 1000 × g for 5 min, and resuspended in sterile PBS at a density of 5 × 10^7^ cells/mL. Each mouse received a subcutaneous injection of 100 μL cell suspension (2 × 10^6^ cells) into the right axillary region.

When tumor volumes reached approximately 100 mm^3^, mice were randomly assigned (random number table method) to three groups: Control (saline), CUR (500 mg/kg CUR), and CUR + LGG (combined CUR and *Lactobacillus rhamnosus* GG) treatment groups. CUR was suspended in 0.5% sodium carboxymethyl cellulose (C104984, Aladdin, China). LGG (1 × 109 CFU/mouse) was cultured anaerobically in MRS medium at 37°C for 24 hours, centrifuged (5000 × g, 10 min, 4°C), and resuspended in sterile saline (1 × 10^10^ CFU/mL). All treatments were administered by oral gavage (10 μL/g body weight) every 3 days for 21 days.

For the GEM-resistant xenograft experiment, tumor-bearing mice were divided into four groups: Control (saline), GEM (50 mg/kg gemcitabine), CUR+LGG, and GEM+CUR+LGG. GEM was dissolved in saline and administered intraperitoneally at 50 mg/kg every 3 days.

Tumor growth was monitored every 3 days using a digital caliper, and tumor volume (V) was calculated as V = (L × W^2^)/2. At the end of the treatment, mice were euthanized via CO_2_ asphyxiation (1.5 L/min for 5 min), and tumors were excised, photographed, and weighed [37, 38].

### Pharmacokinetics of CUR

GEM-resistant GBC-SD xenograft nude mice were randomized into two groups (n=6): CUR (oral CUR, 500 mg/kg) and CUR+LGG (oral CUR 500 mg/kg plus LGG, 1×10^10^ CFU/mL). Animals were fasted for 12 hours before dosing. Blood samples were obtained from the retro-orbital venous plexus at 0, 0.25, 0.5, 1, 2, 4, 8, 12, and 24 hours after administration and collected into heparinized tubes. Plasma was isolated by centrifugation (3000 rpm, 10 min, 4°C) and stored at -80°C until analysis. For HPLC analysis (Waters e2695, USA), 50 μL plasma was mixed with 200 μL methanol containing rutin (5 μg/mL) as the internal standard. After vortex mixing and sonication in an ice bath for 10 min, samples were centrifuged and the supernatant was subjected to chromatographic analysis. Pharmacokinetic parameters, including maximum plasma concentration (Cmax), time to reach maximum concentration (Tmax), area under the plasma concentration–time curve (AUC), elimination half-life (t½), and mean residence time (MRT), were calculated using DAS 3.0 [39].

### TUNEL Assay for Apoptosis Detection

Paraffin-embedded tumor sections (4 μm) were deparaffinized in xylene (two washes, 10 min each) and rehydrated through a graded ethanol series (100%, 95%, 85%, and 70%, 5 min each). Sections were treated with proteinase K (20 μg/mL; ST533, Beyotime) at 37°C for 20 min and rinsed with PBS, and incubated with TUNEL reaction mixture (C1086, Beyotime) at 37°C for 60 min in the dark. Nuclei were counterstained with 1 μg/mL DAPI for 5 min, and slides were mounted with an antifade mounting medium (P0126, Beyotime). Apoptotic cells were visualized under a fluorescence microscope, with five random fields per section analyzed using ImageJ software to quantify the apoptosis rate.

### Quantitative Analysis of Fecal Microbiota by qPCR

Fresh fecal samples were obtained from mice 24 hours before the end of treatment. Microbial genomic DNA was isolated using the QIAamp DNA Stool Mini Kit (51604, Qiagen, Germany). qPCR was performed in a 25 μL reaction system containing 10 μL SYBR Premix Ex Taq II (RR820A, Takara), 0.4 μM specific primers, and 1 μL DNA template. Specific primers targeting LGG, *Bifidobacterium lactis* (BA), *Lactobacillus acidophilus* (LA), Shiga toxin-producing *Escherichia coli*, and *Bacillus cereus* (BC) were synthesized by Sangon Biotech (China) (primer sequences listed in Table S3). Each sample was analyzed in triplicate, and relative abundance was calculated using the 2^-ΔΔCt^ method.

As a targeted technique, qPCR quantifies specific bacterial taxa but does not provide a comprehensive assessment of gut microbiota composition, diversity, or microbial interaction networks. Therefore, the associations between changes in microbial abundance and functional metabolic outcomes require further validation through integrated metagenomic and metabolomic analyses, as well as functional studies such as fecal microbiota transplantation (FMT).

### SCFA Quantification

Fecal samples were collected from mice and thawed at 4°C. Approximately 20 mg of each sample was extracted with 800 μL of 0.5% phosphoric acid containing 2-ethylbutyric acid (10 μg/mL; B65222, SyyBio, China) as an internal standard. After homogenization at 4°C for 3 min (50 kHz) and sonication for 10 min (40 kHz), the suspension was centrifuged (13,000 rpm, 15 min, 4°C). The resulting supernatant was mixed with 200 μL n-butanol, vortexed briefly, sonicated for 10 min, and centrifuged again under the same conditions. The final supernatant was used for subsequent analysi. SCFAs were quantified using a gas chromatography–mass spectrometry (GC–MS) system (7890B/5977B, Agilent, USA) equipped with an HP-FFAP capillary column (30 m × 0.25 mm × 0.25 μm). Helium served as the carrier gas at 1.0 mL/min. Samples (1 μL) were injected with a split ratio of 10:1, and the injector temperature was set to 180°C. The oven program started at 80°C, increased to 120°C at 20°C/min, then to 160°C at 5°C/min, and finally to 220°C at 20°C/min with a 2-min hold. The ion source temperature was maintained at 230°C. Acetic acid, propionic acid, and butyric acid were quantified in MRM mode. SCFA concentrations were expressed as μg/mg (wet weight) for fecal samples and as μM for serum samples [40].

### SCFA Supplementation Experiment

To determine the contribution of SCFAs to the combined intervention, a dedicated SCFA-supplemented group was included in the GEM-resistant xenograft model. Mice received drinking water supplemented daily with a mixture of sodium acetate (67.5 mM), sodium butyrate (40 mM), and sodium propionate (25.9 mM) (Sigma-Aldrich) for 3 weeks. Water intake was monitored daily. Control mice received sodium-matched drinking water. At the study endpoint, mice were sacrificed and tumor tissues, feces, and serum samples were harvested for downstream analyses [41].

### Serum Bile Acid Metabolite Analysis

Blood samples obtained from treated mice were centrifuged (3500 × g, 10 min) to isolate serum, which was stored at -80°C prior to analysis. For metabolite extraction, serum was mixed with an equal volume of methanol to precipitate proteins, followed by vortexing and centrifugation at 4°C for 10 min. The supernatant was filtered through a 0.22 μm membrane. The analytes included the primary bile acids glycocholic acid (GCA) and chenodeoxycholic acid (CDCA), as well as the secondary bile acids DCA and LCA. Separation was achieved using a Waters ACQUITY UPLC BEH C18 column (2.1 × 50 mm, 1.7 μm) with mobile phase A (water + 0.1% formic acid) and B (acetonitrile + 0.1% formic acid) in gradient elution mode. Detection was performed in MRM mode, with quantification based on standard curves established for each bile acid.

### Immunohistochemical (IHC) Analysis

Tumor tissues were fixed in 4% PFA, processed through graded ethanol dehydration, and embedded in paraffin. After deparaffinization and rehydration, endogenous peroxidase activity was blocked using 3% hydrogen peroxide (P0100A, Beyotime). Antigen retrieval was subsequently performed, followed by overnight incubation at 4°C with primary antibodies against phosphorylated PI3K (p-PI3K; PA5-104853, 1:100, Thermo Fisher) and phosphorylated AKT (p-AKT; PA5-36780, 1:100, Thermo Fisher). The following day, HRP-conjugated secondary antibody (A0208, Beyotime) was applied for 1 hours at ambient temperature. Immunoreactivity was visualized with 3,3’-diaminobenzidine (DAB; P0202, Beyotime) and counterstained with hematoxylin (C0107, Beyotime). Images were captured using a bright-field microscope under identical settings for all samples.

### Prediction of Potential CUR Targets

Potential targets of CUR were predicted through database mining approaches. First, 68 CUR-related targets were obtained from the Drug–Gene Interaction Database (DGIdb; data downloaded in June 2025) (Table S4). The chemical structure of CUR was obtained from PubChem (https://pubchem.ncbi.nlm.nih.gov/). Subsequently, the SMILES structure of CUR (COc1cc(/C=C/C(=O)CC(=O)/C=C/c2ccc(c(c2)OC)O)ccc1O) was submitted to SwissTargetPrediction (http://www.swisstargetprediction.ch/) with species set to Homo sapiens, yielding 112 predicted targets (Table S5). All predicted proteins were mapped to official gene symbols using the UniProt database (https://www.uniprot.org/), and redundant entries were removed to obtain a final set of 266 unique coding genes.

### Screening of Drug Resistance-Related Genes in GBC

Drug resistance-related genes in GBC were screened by searching the GeneCards database (https://www.genecards.org/, data retrieved June 2025) and OMIM database (Online Mendelian Inheritance in Man, https://omim.org, data retrieved June 2025) using the keyword “gallbladder cancer drug resistance”. From GeneCards, genes with a relevance score greater than 10 were selected, yielding 1,077 candidates. From OMIM, 201 non-redundant genes were obtained after removing duplicates. The gene lists from both databases were subsequently merged for further analysis.

### Gene Ontology (GO)/Kyoto Encyclopedia of Genes and Genomes (KEGG) Functional Enrichment Analysis

The intersection genes were subjected to GO and KEGG pathway enrichment analyses using the Xiantao Academic online platform. GO enrichment analysis was conducted across three domains, including Biological Process (BP), Cellular Component (CC), and Molecular Function (MF). KEGG pathway analysis was performed using default parameters. *P*-values and q-values were corrected using the Benjamini–Hochberg method, with significance thresholds set at *p* < 0.01 and a minimum overlapping gene count of three. Enrichment results were visualized as bubble plots.

### Protein-Protein Interaction (PPI) Network Construction

The overlapping genes between predicted CUR targets and GBC drug resistance–related genes were uploaded to the STRING database (https://string-db.org) to construct a PPI network. The analysis parameters were set as follows: organism = Homo sapiens and minimum required interaction score = 0.900 (highest confidence), with disconnected nodes removed. The resulting interaction data were exported in TSV format and visualized in Cytoscape (version 3.5.1) for network topology analysis. Hub genes within the network were identified using the CytoHubba plugin, with gene significance ranked according to Degree centrality.

### Statistical Analysis

All data were obtained from at least three independent experiments and are presented as mean ± standard deviation (SD). Differences between two groups were assessed using independent-sample *t* tests, whereas one-way analysis of variance (ANOVA) was used for multiple-group comparisons. When ANOVA indicated significant variation, Tukey’s honest significant difference (HSD) test was performed for post hoc analysis. For data not conforming to normality or homogeneity of variance, nonparametric tests (Mann–Whitney *U* test or Kruskal–Wallis *H* test) were applied. Statistical analyses were carried out using GraphPad Prism 9.5.0 (GraphPad Software, USA) and R 4.2.1 (R Foundation for Statistical Computing, Austria). A two-sided *p*-value < 0.05 was considered statistically significant.

## Results

### Successful Extraction and Identification of High-Purity CUR

**Fig. 1:**
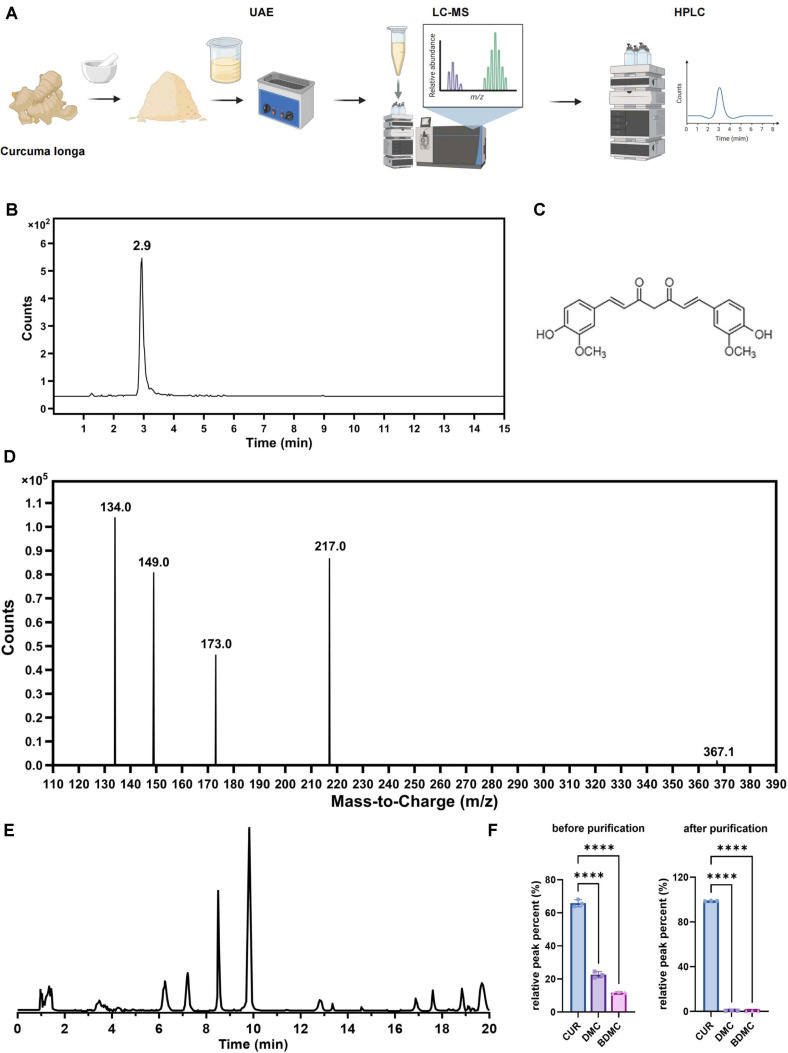
Extraction, purification, and compositional characterization of CUR. (**A**) Schematic workflow of CUR extraction and purification procedures designed using BioRender. (**B**) Purity assessment of CUR extracts by HPLC. (**C**) Molecular structure diagram of CUR. (**D**) Molecular ion peak identification (m/z = 367.1 [M–H]^-^) through LC-MS analysis. (**E**) Extracted ion chromatogram of the crude extract. (**F**) Relative content of three curcuminoids before and after purification. Data are presented as mean ± standard deviation (Mean ± SD), with n = 3 independent extraction and purification experiments. **p < 0.01, ***p < 0.001.

To obtain high-purity CUR for subsequent experiments, CUR was extracted from *Curcuma longa* rhizomes using UAE, followed by purification and characterization (Fig. 1A). The crude extract was first concentrated by rotary evaporation and subjected to LC–MS for preliminary component identification. The chromatographic profile showed that the dominant peak corresponded to the retention time of the CUR reference standard. Further purification using HPLC yielded a single dominant peak in the chromatogram, indicating that the purity of CUR exceeded 98.5% (Fig. 1B).

Using LC–MS in negative ion mode (ESI^-^), the deprotonated [M–H]^-^ peak of CUR was detected at m/z 367.1184 (theoretical C_21_H_19_O_6_^−^: 367.1188), with a mass error of -1.1 ppm, consistent with the molecular formula C_21_H_19_O_6_ (Fig. 1C). No significant formate adducts ([M+HCOO]^-^) or interfering peaks were detected, suggesting high specificity and stability of the analytical method (Fig. 1D).

To comprehensively characterize the extract composition, we further analyzed crude and purified products by LC–MS. Extracted ion chromatograms revealed clear peaks for CUR ([M–H]^-^ m/z 367.1, retention ~9.8 min), DMC ([M–H]^-^ m/z 337.1, ~8.5 min), and BDMC ([M–H]^-^ m/z 307.1, ~7.2 min) in the crude extract (Fig. 1E). Semi-quantitative analysis indicated that DMC and BDMC accounted for approximately 22.5% and 11.6% of the crude extract, respectively. After HPLC purification, the relative content of CUR increased markedly to approximately 98.8%, whereas the proportions of DMC and BDMC decreased to below 1% (Fig. 1F). These results indicate that a highly purified CUR monomer was successfully obtained for subsequent cellular and animal experiments.

### Construction and Validation of GEM-Resistant Cell Line

To establish a stable GEM-resistant model, GBC-SD cells were continuously exposed to gradually increasing concentrations of GEM over approximately 20 passages. This process generated a resistant subline capable of stable proliferation in the presence of 5 μM GEM, designated GBC-SD/GEM. To validate the resistant phenotype, we measured GEM dose–response curves of parental and resistant cells using the CCK-8 assay. Parental cells exhibited an IC_50_ of 1.2 ± 0.3 μM, whereas the resistant line showed a markedly elevated IC_50_ of 8.6 ± 0.9 μM (Fig. S1), giving a RI of 7.17. These results confirm the successful construction of a robust GEM-resistant model for subsequent reversal experiments.

### Synergistic Inhibition of GBC Cell Proliferation and Migration and Induction of Apoptosis by CUR Combined with Probiotics

**Fig. 2:**
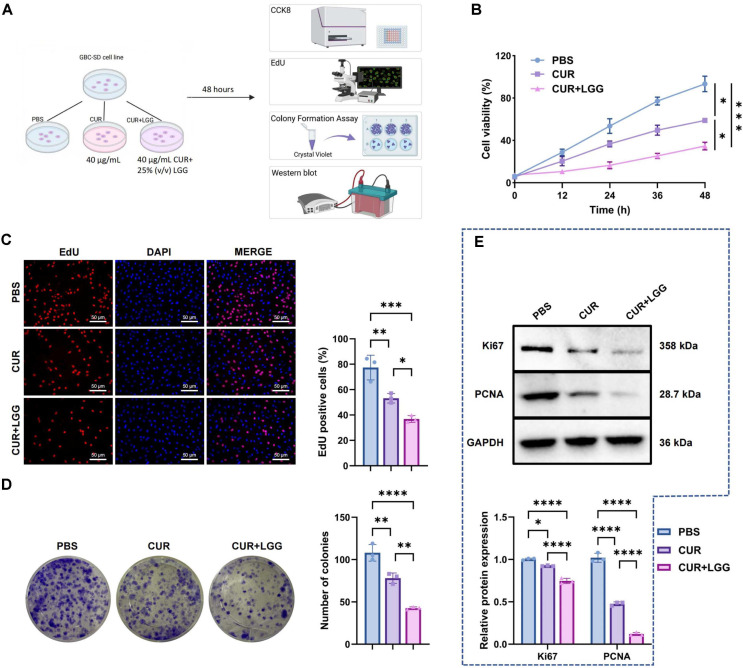
Inhibitory effects of CUR-LGG combination on GBC-SD cell proliferation. (**A**) Schematic diagram of the experimental grouping showing that human GBC GBC-SD cells were treated with PBS (control), 40 μg/mL curcumin (CUR), or 40 μg/mL curcumin combined with 25% (v/v) LGG cell-free supernatant (CUR+LGG), Created in BioRender, (**B**) CCK-8 assay detecting the changes in viability of GBC-SD cells in different treatment groups (48 hours treatment). (**C**) EdU staining assessing the proliferation of GBC-SD cells in different treatment groups (red indicates EdU-positive signals, 24 h treatment) (scale bar = 25 μm). (**D**) Colony formation assay evaluating the colony-forming ability of GBC-SD cells in different treatment groups (10 days treatment) (scale bar = 50 μm). (**E**) Western blot analysis detecting the protein expression levels of Ki67 and PCNA in GBC-SD cells in different treatment groups (48 h treatment). n = 3. Data are presented as mean ± standard deviation (Mean ± SD). *p <0.05, ***p <0.001, ****p <0.0001.

Next, we investigated the combined effects of the extracted CUR and LGG on the biological characteristics of GBC-SD cells (Fig. 2A). CCK-8 analysis indicated that CUR treatment decreased cell viability to approximately 63% of the control level, whereas CUR+LGG co-treatment further decreased viability to about 37% (Fig. 2B). Consistently, EdU staining revealed a marked reduction in proliferative activity. The proportion of EdU-positive cells decreased from approximately 77% in the control group to 53% after CUR treatment and was further reduced to about 37% following CUR+LGG co-treatment (Fig. 2C). Colony formation assays showed similar results, with CUR significantly inhibiting clonogenic growth compared with the control group, while the combination with LGG resulted in a more pronounced suppression of colony formation (Fig. 2D). WB analysis showed that CUR treatment decreased Ki67 and PCNA protein levels by approximately 8% and 53%, respectively. The combination of CUR and LGG further suppressed their expression to about 74% and 12% of the control levels (Fig. 2E).

**Fig. 3:**
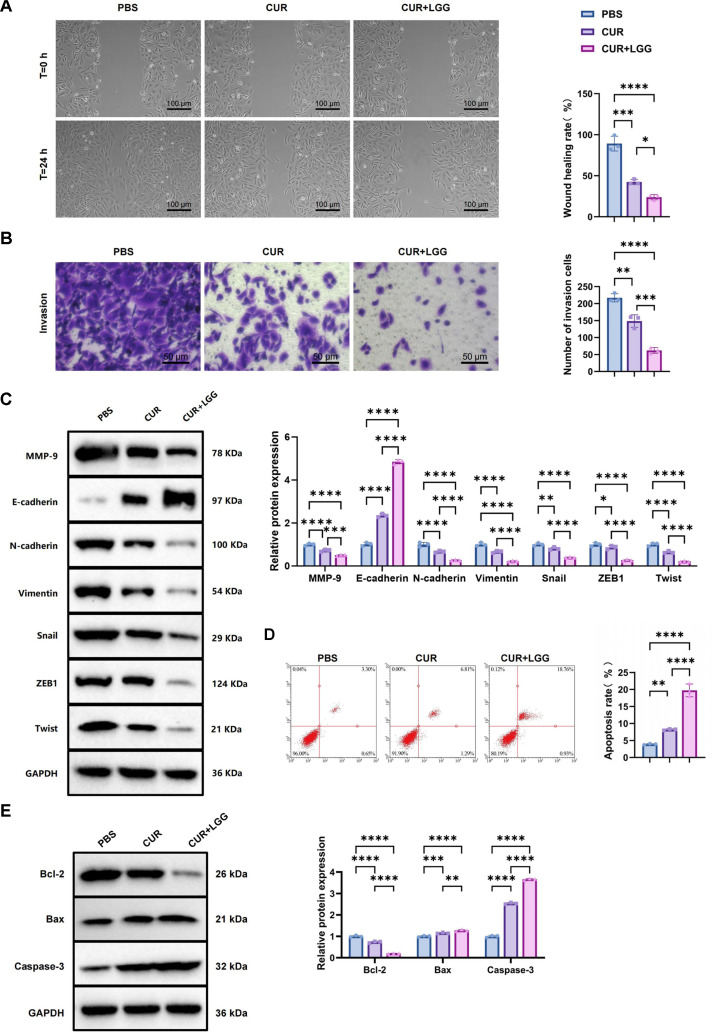
Effects of CUR combined with LGG intervention on migration, invasion and apoptosis in GBC-SD cells. (**A**) Cell migration capacity assessed by wound healing assay (scale bar: 100 μm). (**B**) Cell invasion ability evaluated by Transwell assay (scale bar: 50 μm). (**C**) Protein expression levels of migration/invasion-related markers (MMP-9 and E-cadherin) detected by Western blot; (**D**) Apoptotic cell ratio measured by flow cytometry; (**E**) Expression levels of apoptosis-related proteins (Bcl-2, Bax and Caspase-3) analyzed by Western blot. n = 3. Data are presented as mean ± standard deviation (Mean ± SD). *p <0.05, **p < 0.01, ***p < 0.001, ****p < 0.0001.

Cell migration and invasion were first evaluated using wound healing and Transwell assays. In the wound healing assay, the wound closure rate in the control group reached approximately 89%. CUR treatment significantly reduced the closure rate to about 42%, while CUR+LGG co-treatment further suppressed migration to approximately 24% of the control level (Fig. 3A). Consistently, the Transwell invasion assay showed that CUR inhibited invasive capacity, whereas the CUR+LGG combination produced a more pronounced reduction in the number of invading cells compared with CUR alone (Fig. 3B). WB analysis further confirmed these changes at the molecular level. CUR treatment upregulated the epithelial marker E-cadherin while suppressing mesenchymal markers, including N-cadherin and Vimentin, as well as the epithelial–mesenchymal transition (EMT)-associated transcription factors Snail and ZEB1. Co-treatment with LGG further enhanced E-cadherin expression and produced stronger suppression of N-cadherin, Vimentin, Snail, Twist, and ZEB1 compared with CUR monotherapy (Fig. 3C). Apoptosis analysis by flow cytometry showed that CUR induced an apoptosis rate of approximately 8.2%, which increased to about 19.7% after CUR+LGG co-treatment (Fig. 3D). WB analysis demonstrated that CUR treatment reduced Bcl-2 expression by approximately 27%, while the expression of Bax and cleaved Caspase-3 increased by about 1.15-fold and 2.5-fold, respectively. These changes were more pronounced after CUR+LGG co-treatment, with Bcl-2 expression decreasing to about 20% of the control level, whereas Bax and cleaved Caspase-3 expression increased to approximately 1.28-fold and 3.67-fold of the control levels, respectively (Fig. 3E).

In summary, CUR markedly suppressed the malignant phenotypes of GBC cells, including reduced cell growth and motility, while promoting apoptosis. Co-treatment with LGG further enhanced these effects, suggesting a synergistic role of the CUR–LGG combination in regulating GBC cell behavior.

### CUR Combined with LGG Significantly Suppresses GBC Growth in Mice

**Fig. 4:**
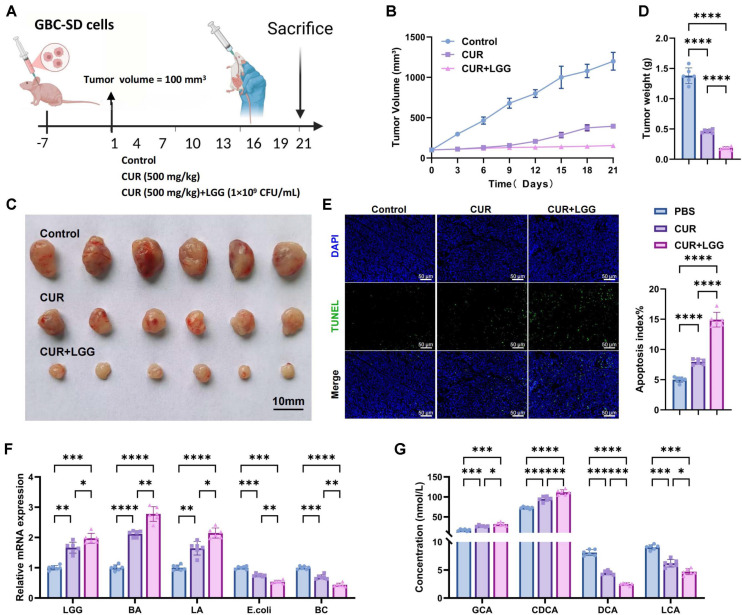
Effects of CUR combined with LGG intervention on GBC-bearing mice. (**A**) Schematic diagram of the GBC-SD tumor-bearing mouse model and intervention protocol. Mice were randomly divided into three groups (n = 6 per group): Control group (0.5% sodium carboxymethyl cellulose administered by gavage), CUR group (curcumin 500 mg/kg by gavage), and CUR+LGG group (curcumin 500 mg/kg + LGG 1×10^9^ CFU/mouse by gavage). All gavage interventions were administered once every 3 days for 21 days. Created in BioRender. (**B**) Tumor volume growth curves during treatment. (**C**) Representative photographs of excised tumor tissues at the end of treatment. (**D**) Tumor weight measurements. (**E**) TUNEL staining showing apoptotic cells (green: TUNEL-positive, blue: DAPI; scale bar: 50 μm). (**F**) qPCR analysis of fecal microbiota composition (LGG, BA, LA, *E. coli*, BC). (**G**) LC-MS analysis of the serum concentrations of glycocholic acid (GCA), chenodeoxycholic acid (CDCA), deoxycholic acid (DCA), and lithocholic acid (LCA). n = 6. Data are presented as mean ± standard deviation (Mean ± SD). *p < 0.05, **p < 0.01, ***p < 0.001, ****p < 0.0001.

To systematically evaluate the inhibitory effects of CUR combined with LGG on tumor growth, we established a subcutaneous xenograft model of GBC (GBC-SD) in mice (Fig. 4A). After three weeks of oral administration, tumor growth was assessed. CUR treatment significantly decreased tumor volume and weight relative to the control group. The addition of LGG further strengthened this antitumor effect, producing greater inhibition of tumor growth than CUR alone (Fig. 4B-4D). TUNEL staining revealed increased apoptotic signals in tumor tissues after CUR treatment, which were further elevated in the CUR+LGG group (Fig. 4E). These results indicate that the antitumor effect of the combined treatment may be associated with enhanced apoptosis.

To explore whether the treatment influenced gut microbial composition, qPCR was performed to assess the abundance of selected bacterial taxa in fecal samples. CUR treatment markedly increased the abundance of beneficial bacteria, including LGG, BA, and LA, while decreasing pathogenic species such as *Escherichia coli* and BC relative to the control group. Importantly, the CUR+LGG combination further enhanced probiotic abundance and produced stronger suppression of pathogenic bacteria compared with CUR alone (Fig. 4F).

Bile acid metabolic profiles were subsequently analyzed by LC–MS. CUR treatment significantly increased the levels of primary bile acids, including GCA and CDCA while decreasing the levels of secondary bile acids such as DCA and LCA. The CUR+LGG combination further amplified these metabolic changes, showing greater elevation of primary bile acids and stronger suppression of secondary bile acids (Fig. 4G). Collectively, these findings indicate that CUR combined with LGG not only suppresses tumor growth but is also linked to coordinated alterations in gut microbial composition and bile acid metabolism. The observed reductions in secondary bile acids (DCA and LCA) represent functional metabolic outcomes reflecting changes in bile acid metabolism; however, the direct causal relationships between specific microbial taxa and these metabolic alterations require further investigation.

Collectively, our findings demonstrate that the combined intervention with CUR and LGG can effectively ameliorate bile acid metabolic imbalance by regulating the abundance of specific gut bacteria and modulating bile acid metabolism, thereby promoting apoptosis in GBC cells and ultimately inhibiting tumor growth.

### LGG Synergistically Enhances the *In Vivo* Bioavailability of CUR

To evaluate whether LGG enhanced the antitumor efficacy of CUR by improving its pharmacokinetic behavior, plasma drug concentration profiles were compared between CUR monotherapy and CUR+LGG treatment groups. The CUR+LGG group exhibited consistently higher plasma CUR concentrations at all sampling points relative to CUR alone (Fig. S2, Table S6). Quantitative pharmacokinetic analysis revealed that co-administration with LGG increased the maximum plasma concentration (Cmax) by 1.44-fold and the area under the concentration–time curve (AUC) by 3.06-fold, indicating markedly enhanced systemic exposure and bioavailability. In addition, the elimination half-life (T1/2) and MRT of CUR were significantly prolonged in the combination group, suggesting reduced metabolic clearance. These findings indicate that LGG substantially improves the *in vivo* bioavailability of CUR, providing an important pharmacokinetic basis for the enhanced antitumor activity and chemoresistance-reversal effects observed with the CUR+LGG combination.

### Synergistic Reversal of GEM Resistance by CUR and Probiotic Combination Therapy in GBC

**Fig. 5:**
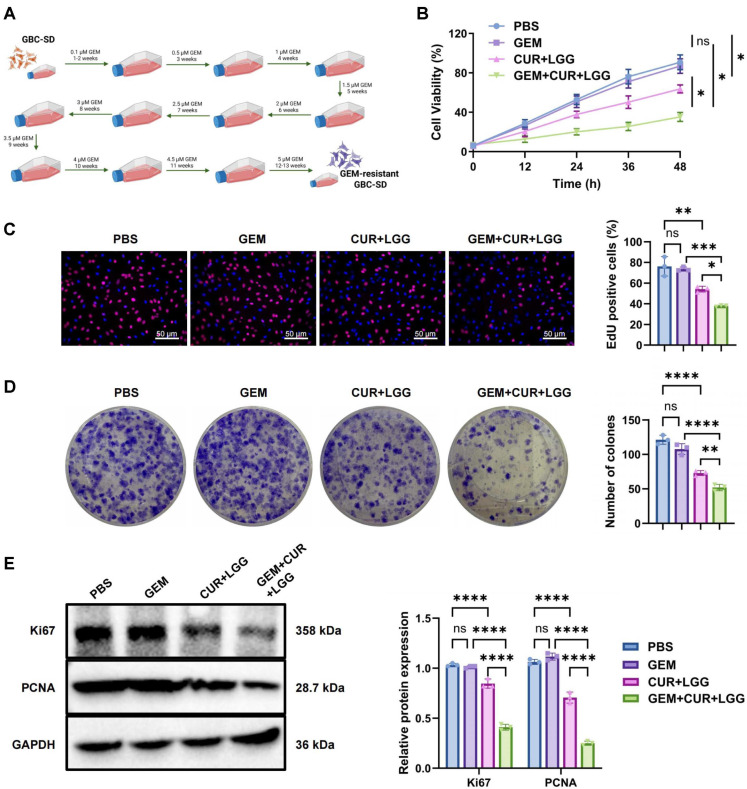
Inhibitory effects of CUR-LGG combination on proliferation of GEM-resistant GBC cells. (**A**) Schematic diagram of the treatment procedure for GEM-resistant GBC-SD cells. Treatment groups included PBS, GEM (5 μM), CUR+LGG (40 μg/mL CUR + 25% v/v LGG supernatant), and GEM+CUR+LGG. Cells were treated for 48 h (Created in BioRender). (**B**) Cell viability measured by CCK-8 assay. (**C**) Cell proliferation assessed by EdU staining (red: EdU-positive cells, blue: DAPI nuclear staining; scale bar: 50 μm). (**D**) Colony formation capacity evaluated by clonogenic assay; (**E**) Protein expression levels of proliferation markers (Ki67 and PCNA) determined by Western blot analysis. n = 3. Data are presented as mean ± standard deviation (Mean ± SD). *p < 0.05, **p < 0.01, ***p < 0.001, ****p < 0.0001. ns: not statistically significant.

We established GEM-resistant GBC-SD GBC cell lines to systematically evaluate the effects of CUR-probiotic combination therapy on drug-resistant cell characteristics (Fig. 5A). CCK-8 assays showed that GEM treatment alone had minimal impact on cell viability. In contrast, CUR+LGG treatment reduced viability to approximately 70% of the control level, while the triple combination (GEM+CUR+LGG) further decreased viability to about 39% (Fig. 5B). EdU staining confirmed reduced proliferative activity following CUR+LGG treatment, with the GEM+CUR+LGG combination producing a more pronounced decrease in EdU-positive nuclei (Fig. 5C). Similarly, colony formation assays demonstrated fewer colonies in the CUR+LGG group compared with the PBS control, and an additional reduction was observed in the triple-combination group (Fig. 5D). WB analysis confirmed the downregulation of Ki67 and PCNA protein expression in the CUR+LGG group, with the GEM+CUR+LGG combination further significantly suppressing these proliferation markers (Fig. 5E).

**Fig. 6:**
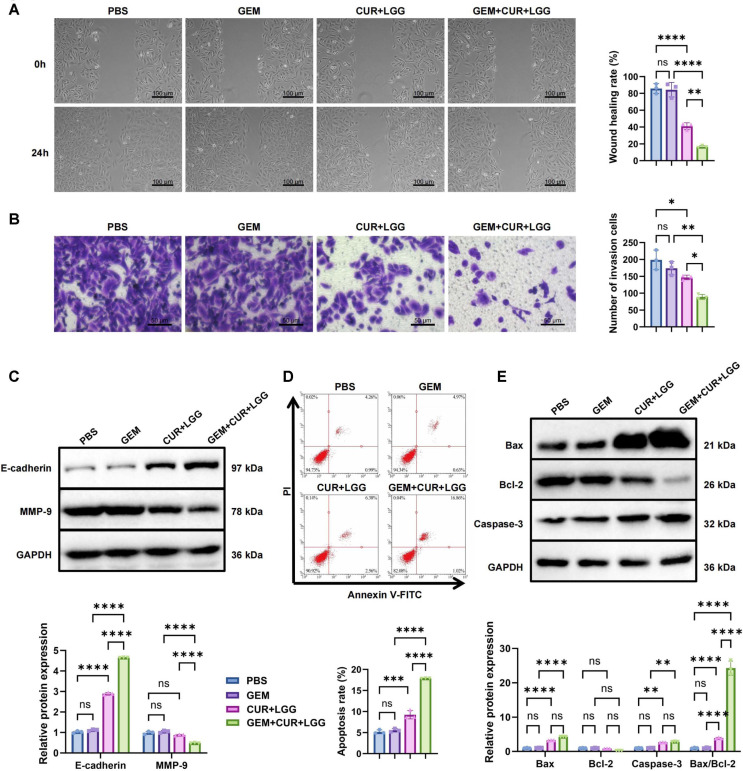
Effects of CUR combined with LGG intervention on migration, invasion and apoptosis in GEM-resistant GBC cells. (**A**) Cell migration ability detected by wound healing assay (scale bar: 100 μm). (**B**) Cell invasion capacity evaluated by Transwell assay (scale bar: 50 μm). (**C**) Western blot analysis of migration/invasion-related proteins (MMP-9 and E-cadherin). (**D**) Apoptosis rate measured by flow cytometry. (**E**) Western blot detection of apoptosis-related proteins (Bcl-2, Bax and Caspase-3). n = 3. Data are presented as mean ± standard deviation (Mean ± SD). *p < 0.05, **p < 0.01, ***p < 0.001, ****p < 0.0001. ns: no significant difference.

In cell migration and invasion assays, the scratch wound healing test demonstrated significantly inhibited cell migration in the CUR+LGG group, with the GEM+CUR+LGG combination showing a further reduction in migration distance (Fig. 6A). Consistently, Transwell assays showed fewer invading cells in the CUR+LGG group, with the GEM+CUR+LGG treatment producing the strongest suppression of invasive capacity (Fig. 6B). WB analysis revealed downregulation of MMP-9 and upregulation of E-cadherin in the CUR+LGG group, with the GEM+CUR+LGG combination showing additional significant modulation of these EMT markers (Fig. 6C). Apoptosis analysis by flow cytometry showed that CUR+LGG treatment induced an apoptosis rate of approximately 9.25%, which increased markedly to 17.87% after the addition of GEM (Fig. 6D). WB results further confirmed that Bcl-2 expression was downregulated and Bax and cleaved Caspase-3 were upregulated in the CUR+LGG group, with the Bax/Bcl-2 ratio increasing to 3.91 ± 0.16. These apoptosis-related changes became more pronounced in the GEM+CUR+LGG group, where the Bax/Bcl-2 ratio reached its highest level at 24.27 ± 2.07 (vs. PBS group 0.99 ± 0.12, *p* < 0.001) (Fig. 6E). The marked increase in the Bax/Bcl-2 ratio provides molecular evidence that the combined treatment effectively reverses GEM resistance and robustly activates the apoptotic signaling cascade, reflecting enhanced chemosensitivity [42, 43].

In summary, GEM-resistant GBC-SD cells exhibited limited responsiveness to GEM monotherapy, whereas the combination of CUR and LGG markedly suppressed malignant cellular behaviors and promoted apoptotic signaling. Notably, the triple combination (GEM+CUR+LGG) produced the strongest inhibitory effects, highlighting a synergistic therapeutic potential and suggesting that the CUR–probiotic strategy may represent a promising approach for overcoming chemoresistance in GBC.

### CUR Combined with LGG Reverses GEM Resistance and Modulates Gut Microbiota in GBC

**Fig. 7:**
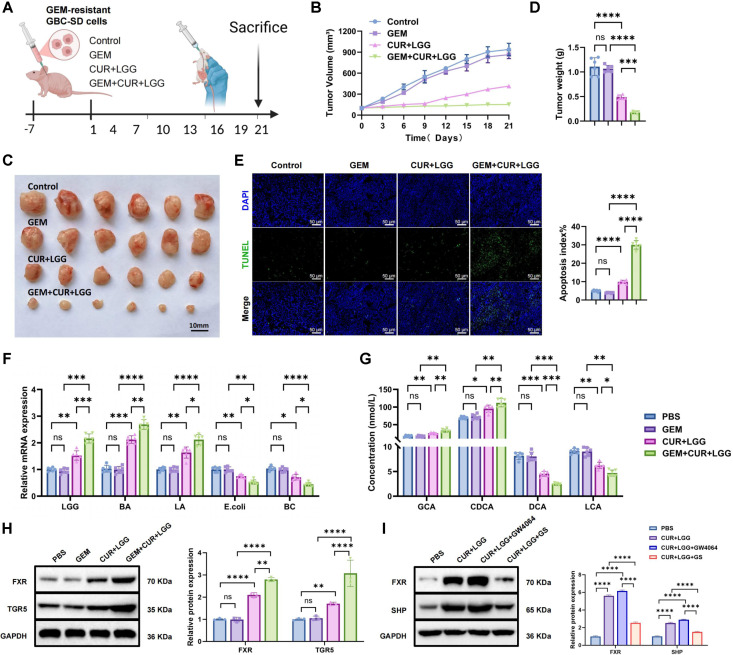
Therapeutic effects of CUR-LGG combination on GEM-resistant GBC xenografts. (**A**) Schematic diagram of the intervention protocol for GEM-resistant GBC-SD tumor-bearing mice. Mice were randomly divided into four groups (n = 6 per group): Control group (vehicle control), GEM group (gemcitabine 50 mg/kg intraperitoneal injection), CUR+LGG group (curcumin and LGG administered by gavage, same dosage as previously described), and GEM+CUR+LGG group (gemcitabine intraperitoneal injection + curcumin and LGG gavage). Gemcitabine was administered intraperitoneally once every 3 days for a total intervention period of 21 days (Created in BioRender). (**B**) Tumor growth curves during treatment period. (**C**) Macroscopic appearance of excised tumors at endpoint (scale bar: 10 mm). (**D**) Tumor weight measurements at study termination. (**E**) TUNEL staining of tumor sections showing apoptotic cells (green: TUNEL-positive, blue: DAPI; scale bar: 50 μm). (**F**) qPCR quantification of fecal microbiota (LGG, BA, LA, *E. coli*, BC). (**G**) LC-MS analysis of serum concentrations of GCA, CDCA, DCA, and LCA;. (**H**) Western blot analysis of FXR and TGR5 protein levels in tumor tissues of mice; (**I**) Effects of FXR agonist and antagonist on CUR+LGG-induced FXR/SHP signaling *in vitro*. n = 6. Data are presented as mean ± standard deviation (Mean ± SD). *p < 0.05, **p < 0.01, ***p < 0.001, ****p < 0.0001. ns: no significant difference.

To systematically evaluate the efficacy of CUR combined with probiotics in overcoming chemotherapy resistance *in vivo*, we established a subcutaneous xenograft model using GEM-resistant GBC-SD cells in nude mice (Fig. 7A). Tumor growth was monitored every three days during the three-week treatment period. GEM monotherapy showed no significant inhibition of tumor growth compared with the control group. In contrast, CUR+LGG treatment reduced tumor volume by approximately 56%, whereas the triple regimen (GEM+CUR+LGG) produced a stronger synergistic effect, decreasing tumor volume by approximately 84% (Fig. 7B). Consistent with these findings, final tumor weight measurements showed significant reductions in the CUR+LGG group and an additional decrease in the GEM+CUR+LGG group (Fig. 7C and 7D). TUNEL staining revealed no significant difference in apoptosis between the GEM and Control groups. In contrast, the CUR+LGG group displayed markedly enhanced TUNEL-positive signals in tumor tissues, and the GEM+CUR+LGG combination further significantly increased apoptotic activity (Fig. 7E).

To determine whether the combined treatment influenced gut microbial composition, fecal bacterial abundance was assessed by qPCR. No significant differences were observed between the GEM and control groups. However, both the CUR+LGG and GEM+CUR+LGG treatments significantly increased beneficial bacteria (LGG, *Bifidobacterium*, and *Lactobacillus*) while reducing pathogenic taxa (*E. coli* and *Clostridium*) (Fig. 7F). These findings indicate that the CUR–probiotic intervention reshapes the abundance of several key bacterial populations. Notably, as a targeted method, qPCR reflects relative changes in selected taxa rather than the overall microbial community structure.

Given the close relationship between microbial metabolism and tumor progression, fecal SCFAs were further quantified. GC–MS analysis revealed that acetate, propionate, and butyrate levels were elevated in both the CUR+LGG and GEM+CUR+LGG groups, with butyrate showing the most pronounced increase (Fig. S3A–S3C). To evaluate whether SCFAs contributed to the antitumor effect, an SCFA supplementation experiment was conducted. Mice receiving SCFA-enriched drinking water exhibited reduced tumor volume and weight, accompanied by increased TUNEL-positive apoptotic signals (Fig. S3D–S3F). These results suggest that SCFAs partially mimic the tumor-suppressive effects of LGG.

Serum bile acid metabolism was further analyzed by LC–MS to evaluate its role in overcoming drug resistance. The results showed that CUR+LGG intervention increased the levels of the primary bile acids GCA and CDCA in serum by approximately 1.5-fold and 1.4-fold, respectively, while the levels of the secondary bile acids DCA and LCA decreased by approximately 44% and 32%, respectively. The GEM+CUR+LGG group exhibited a similar but more pronounced trend (Fig. 7G). These findings indicate that the CUR–probiotic intervention is associated with coordinated alterations in gut microbial composition and bile acid metabolism. The observed reduction in secondary bile acids represents a functional metabolic outcome reflecting an overall shift in bile acid metabolism; however, the direct contribution of specific bile-acid–producing bacteria requires further validation.

We next examined whether these metabolic changes were associated with activation of bile acid–responsive signaling pathways. WB analysis showed that GEM alone did not significantly affect FXR or TGR5 expression in tumor tissues. In contrast, CUR+LGG treatment markedly increased FXR and TGR5 levels, while the GEM+CUR+LGG regimen produced the strongest activation (Fig. 7H).

To clarify the causal role of FXR activation in reversing chemoresistance, pharmacological validation was conducted in GEM-resistant cells. CUR+LGG treatment significantly upregulated FXR and its downstream target SHP. Addition of the FXR agonist GW4064 further enhanced CUR+LGG-induced FXR/SHP activation, whereas the FXR antagonist guggulsterone (GS) markedly attenuated this effect (Fig. 7I). These findings demonstrate that activation of FXR signaling contributes to the chemoresistance-reversal effect mediated by the CUR–probiotic combination.

In summary, our *in vivo* studies demonstrate that CUR combined with LGG markedly restrains tumor progression, enhances apoptotic activity, rebalances the gut microbiota, and reshapes bile acid metabolism in GEM-resistant models. The enhanced efficacy observed with GEM co-treatment further supports this combinatorial approach as a potential strategy for overcoming chemotherapy resistance in GBC.

### CUR Combined with LGG Significantly Prolongs Survival and Enhances the Sensitization Ratio in GEM-Resistant Tumor-Bearing Mice

To further assess the long-term therapeutic efficacy, survival analysis was performed in tumor-bearing mice. Compared with the Control group, CUR+LGG alone showed a trend toward prolonged survival without statistical significance. Low-dose GEM (GEM-L, 25 mg/kg) alone yielded limited efficacy, with only slight improvement in median survival time versus Control. Strikingly, GEM-L combined with CUR+LGG significantly extended median survival, with survival curves separating from GEM-L monotherapy and showing significant statistical difference (*p* < 0.01). High-dose GEM (GEM-H, 50 mg/kg) alone showed stronger antitumor efficacy; however, the addition of CUR+LGG further improved survival outcomes (*p* < 0.05) (Fig. S4A–S4E).

Quantitative survival analysis demonstrated that the survival fraction on day 40 (SF2) increased from 0.66 in the GEM-L group to 0.71 in the GEM-L+CUR+LGG group. Similarly, SF2 increased from 0.60 in the GEM-H group to 0.71 after combination treatment. The calculated sensitization enhancement ratios (SER) were 1.33 for GEM-L and 1.22 for GEM-H (SER > 1), indicating that CUR+LGG enhanced the therapeutic response to GEM *in vivo* (Table S7).

### CUR Combined with LGG Modulates the Expression of GEM-Resistance–Associated Key Factors hENT1 and P-gp

To elucidate the molecular mechanisms underlying chemosensitization, we examined known GEM resistance markers. GEM uptake depends on human equilibrative nucleoside transporter 1 (hENT1), while efflux is largely mediated by P-glycoprotein (P-gp).

qRT-PCR and WB analyses showed that GEM-resistant GBC-SD cells exhibited significantly decreased hENT1 expression and markedly increased P-gp levels compared with parental cells (Fig. S5A–S5B), consistent with their resistant phenotype. In GEM-resistant cells, CUR+LGG significantly upregulated hENT1 and suppressed P-gp compared with PBS. Notably, GEM+CUR+LGG triple therapy achieved the strongest upregulation of hENT1 and downregulation of P-gp. Similar molecular trends were observed in tumor tissues of GEM-resistant mice, confirming *in vivo* consistency (Fig. S5C–S5F).

These results suggest that CUR+LGG reverses GEM resistance through a dual mechanism—promoting drug uptake by upregulating hENT1 while inhibiting drug efflux via P-gp suppression—thereby enhancing GEM intracellular accumulation and overcoming GBC chemoresistance.

### Bioinformatics Analysis Reveals CUR's Mechanism of Reversing Chemotherapy Resistance in GBC Through PI3K/AKT Pathway Regulation

**Fig. 8:**
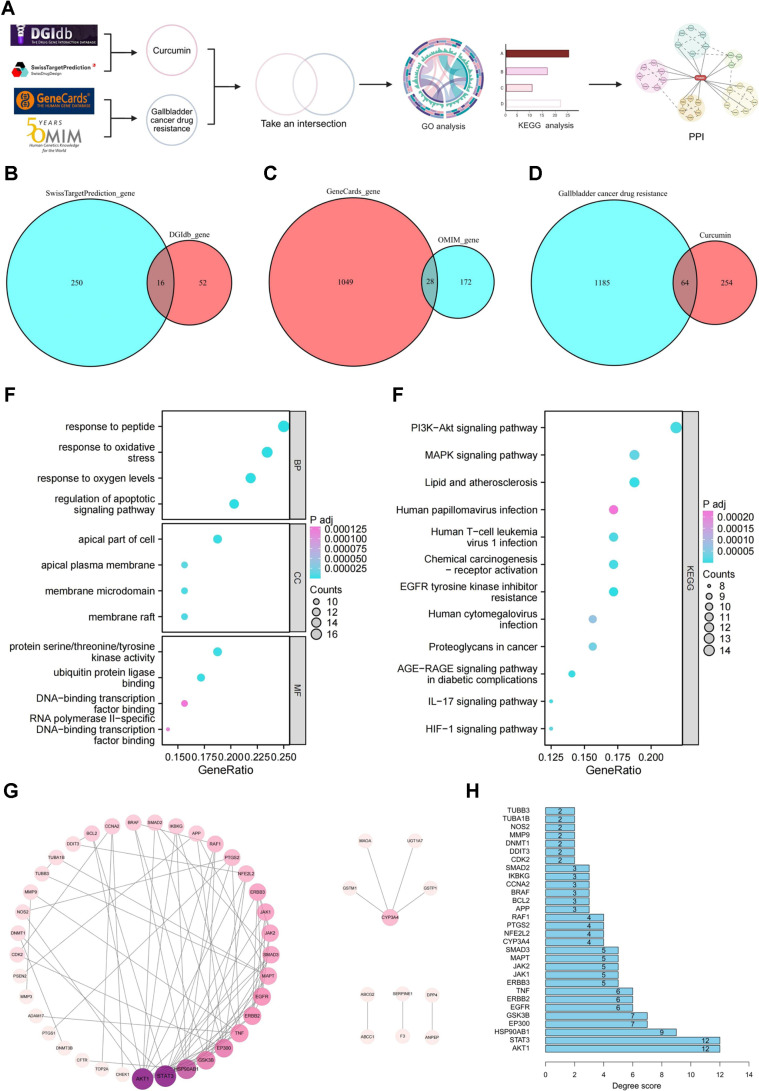
Bioinformatics analysis of potential mechanisms of CUR in GBC chemoresistance. (**A**) Schematic workflow illustrating the identification of overlapping targets between curcumin and gallbladder cancer chemoresistance: curcumin targets were screened using the TCMSP and SwissTargetPrediction databases; chemoresistance-related genes were identified from the GeneCards and OMIM databases; the intersection was visualized to obtain shared targets. Created in BioRender. (**B**) Venn diagram showing the overlapping targets of curcumin identified by the DGIdb and SwissTargetPrediction databases. (**C**) Venn diagram displaying the intersection of gallbladder cancer chemoresistance-related genes identified from the GeneCards and OMIM databases. (**D**) Venn diagram showing the common targets between curcumin-related targets and gallbladder cancer chemoresistance genes. (**E**) GO enrichment analysis of the 64 candidate targets, illustrating significantly enriched terms in BP, MF, and CC. (**F**) KEGG pathway enrichment analysis revealing key signaling pathways through which curcumin may exert its effects on gallbladder cancer chemoresistance. (**G**) PPI network of the 64 candidate targets (Combined score = 0.9); the color gradient from light to dark purple represents increasing Degree values of the genes. (**H**) Statistical analysis of interaction nodes within the PPI network of the 64 candidate targets, displayed in a bar chart.

To systematically elucidate the molecular mechanisms underlying CUR's ability to reverse chemoresistance in GBC, we conducted comprehensive database mining and bioinformatics analysis of CUR-targeted genes and chemoresistance-associated genes (Fig. 8A). Target prediction using DGIdb and SwissTargetPrediction identified 68 and 266 potential CUR-related genes, respectively, yielding 318 unique targets after dataset integration (Fig. 8B). Subsequent analysis of GeneCards and OMIM databases compiled 1,249 GBC chemoresistance-associated genes (Fig. 8C). Intersection analysis of these datasets identified 64 candidate genes potentially involved in the anti-resistance effects of CUR (Fig. 8D).

Functional enrichment analyses were subsequently performed to characterize the biological roles of these candidate targets. GO analysis indicated that the most significantly enriched biological processes included responses to peptides, oxidative stress, and oxygen level changes. Enriched cellular components were mainly related to the apical cell region, apical plasma membrane, and membrane microdomains, whereas molecular functions were primarily associated with protein kinase activity, ubiquitin ligase binding, and transcription factor binding (Fig. 8E). KEGG pathway enrichment further demonstrated that these targets were predominantly involved in the PI3K–AKT signaling pathway, followed by MAPK signaling and pathways related to lipid metabolism and viral infection (Fig. 8F).

To identify key regulatory nodes, a PPI network was constructed using the STRING database (interaction score ≥0.900) and visualized in Cytoscape. Topological analysis using the CytoHubba algorithm highlighted AKT1 and STAT3 as hub genes with the highest degree values (Fig. 8G–8H). These findings indicate that CUR may counteract chemoresistance in GBC primarily through regulation of PI3K/AKT signaling.

In conclusion, our comprehensive bioinformatics analysis demonstrates that CUR reverses chemoresistance in GBC primarily by inhibiting the PI3K/AKT signaling pathway.

### CUR Combined with Probiotics Is associated with Inhibition of the PI3K/AKT Pathway and Reversal of GEM Resistance

**Fig. 9:**
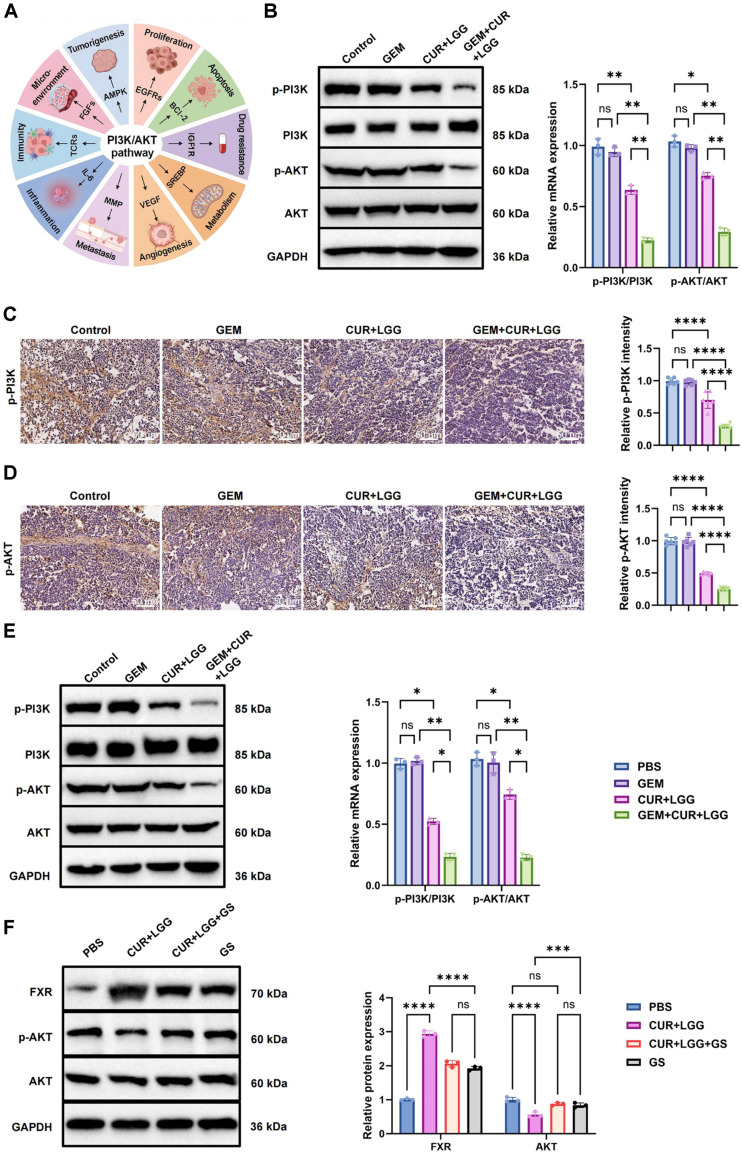
Mechanism of CUR-probiotic combination in reversing GEM resistance via PI3K/AKT pathway inhibition. (**A**) Schematic representation of PI3K/AKT pathway regulation of BP through key mediators (Created in BioRender). (**B**) Western blot analysis of PI3K, AKT, p-PI3K and p-AKT expression in tumor tissues. (**C-D**) IHC staining of p-PI3K and p-AKT in tumor sections (scale bar: 50 μm). (**E**) Western blot detection of PI3K/AKT pathway proteins in GEMresistant GBC-SD cells. (**F**) The effect of the FXR antagonist GS on CUR+LGG-mediated regulation of the p-AKT signaling pathway as detected by Western blot. The number of mice in each group was six (n = 6), and the cell experiments were performed in triplicate (n = 3). Data are presented as mean ± standard deviation (Mean ± SD). *p <0.05, **p < 0.01, ***p < 0.001, ****p < 0.0001. ns: no significant difference.

Previous studies have demonstrated that the PI3K–AKT signaling pathway plays a central role in regulating multiple cellular processes, including cell growth, apoptosis, invasion, differentiation, and metabolism (Fig. 9A) [44].

To verify whether the CUR-probiotic combination could reverse GEM resistance through the PI3K/AKT pathway, we conducted WB and IHC experiments in GEM-resistant GBC-SD xenograft models. WB analysis showed that CUR+LGG treatment significantly inhibited activation of the PI3K/AKT pathway in tumor tissues. Densitometric quantification normalized to GAPDH (n = 3) indicated that phosphorylated AKT (p-AKT) decreased from 100.0 ± 8.5% in the Control group to 72.0 ± 3.1% (*p* < 0.001), while phosphorylated PI3K (p-PI3K) decreased to 68.2 ± 5.4% (*p* < 0.01). The triple combination (GEM+CUR+LGG) produced a more pronounced inhibitory effect, further reducing p-AKT and p-PI3K levels to 25.3 ± 4.8% (*p* < 0.05) and 22.7 ± 3.9% (*p* < 0.01), respectively (Fig. 9B). IHC results showed no significant differences in p-AKT or p-PI3K expression between the GEM and Control groups. In contrast, CUR+LGG treatment markedly reduced the expression of these phosphorylation markers, while the GEM+CUR+LGG regimen produced the strongest suppression of PI3K/AKT pathway activation (Fig. 9C and 9D).

Consistent results were obtained in GEM-resistant GBC-SD cells. WB analysis showed that CUR+LGG treatment markedly reduced p-AKT and p-PI3K expression compared with PBS controls, and the triple combination further enhanced this inhibitory effect (Fig. 9E).

We further examined whether GS affects the CUR+LGG response *in vitro*. Compared with PBS, CUR+LGG markedly increased FXR expression and significantly reduced p-AKT levels. However, addition of GS attenuated the p-AKT suppression by CUR+LGG, partially restoring p-AKT expression. Notably, GS alone had no significant effect on p-AKT. These findings suggest that FXR activation is essential for CUR+LGG to suppress the downstream PI3K/AKT pathway (Fig. 9F).

In summary, the combined intervention of CUR and LGG inhibited the activity of the PI3K/AKT signaling pathway both *in vitro* and *in vivo* and reversed the GEM-resistant phenotype. Pharmacological intervention experiments further demonstrated that activation of FXR is necessary for this process. Collectively, these findings suggest that inhibition of the PI3K/AKT pathway represents a key downstream event in the anti-resistance effect of the combined intervention.

## Discussion

This study represents the systematic evaluation of the combined application of CUR and LGG in inhibiting GEM-resistant GBC cells. Compared with previous studies using CUR alone, the present findings demonstrate that LGG co-administration significantly enhances the inhibitory effect on GEM-resistant GBC-SD cell growth, reducing the IC_50_ value by approximately 40% [45, 46]. The combination treatment also markedly increased apoptotic activity, accompanied by upregulation of Bax and cleaved Caspase-3 and downregulation of Bcl-2. Importantly, unlike the myelosuppression and systemic toxicity commonly associated with conventional chemotherapy, this regimen produced no observable toxicity at the experimental doses used, supporting its potential as a low-toxicity adjuvant therapeutic strategy. The novelty of this study lies in demonstrating that the combination of probiotics and CUR generates a synergistic antitumor effect in GEM-resistant GBC. The findings further suggest that this synergy may be linked to modulation of the gut microenvironment. However, the precise molecular mechanisms underlying this interaction remain to be fully clarified, particularly whether LGG alters the pharmacokinetic profile of CUR through its metabolic products.

Another important observation was the marked suppression of metastatic potential. CUR+LGG treatment significantly reduced the migratory capacity of GEM-resistant cells and showed stronger inhibition than CUR alone. This effect was associated with decreased MMP-9 expression and increased E-cadherin levels, suggesting that the combined intervention may inhibit EMT. Notably, although LGG alone did not significantly suppress invasion, it enhanced the anti-metastatic activity of CUR in Transwell assays. These findings indicate that probiotics may exert indirect antitumor effects by modulating inflammatory signals within the TME. Collectively, this study highlights the potential of microbiota-based strategies as an adjunct approach for limiting GBC metastasis.

This study achieved significant progress in gut microbiota analysis. qPCR results demonstrated that CUR+LGG intervention increased the abundance of beneficial bacteria (*e.g.*, *Lactobacillus*) while significantly suppressing conditional pathogens (*e.g.*, *Bacteroides*) [47, 48]. Although similar microbiota alterations have been reported in colorectal cancer, the present findings extend these observations to GBC chemoresistance models. Notably, unlike antibiotic-based strategies that broadly eliminate microbiota, LGG supplementation may preserve microbial ecological balance while exerting therapeutic effects. However, the present analysis relied primarily on qPCR rather than 16S rRNA sequencing. As a targeted method, qPCR can quantify specific bacterial taxa but cannot comprehensively evaluate the overall composition or diversity of the gut microbiota. Future studies should employ high-throughput sequencing approaches combined with diversity and differential abundance analyses to systematically characterize microbiota alterations associated with chemotherapy resistance reversal. In addition to microbial changes, the present study observed marked reductions in the secondary bile acids DCA and LCA, reflecting a functional shift in bile acid metabolism. Although certain bacterial groups possessing bile salt hydrolase or 7α-dehydroxylase activity are known to participate in secondary bile acid production, a direct causal relationship between specific microbial taxa and the observed metabolic alterations was not established in this study. The reduction in DCA and LCA may instead result from multiple factors, including altered availability of primary bile acid substrates, changes in microbial metabolic activity, or variations in intestinal transit time. Future investigations should integrate metagenomic analysis to identify microbial species carrying relevant metabolic genes. In addition, FMT or colonization with defined bacterial strains in germ-free or antibiotic-treated animal models will be necessary to directly validate the causal link between microbiota alterations and bile acid metabolic phenotypes.

Another important discovery was related to metabolic regulation. LC–MS analysis showed that the combination intervention increased the levels of primary bile acids (GCA and CDCA) while decreasing the secondary bile acids DCA and LCA. This metabolic profile differs from patterns previously reported in obesity-associated liver cancer, suggesting that GBC may possess unique bile acid metabolism characteristics. Importantly, a strong association was observed between the reduction in DCA/LCA levels and inhibition of the PI3K/AKT pathway. Pharmacological validation using an FXR antagonist further indicated that bile acid receptor signaling contributes to this regulatory process. WB analysis *in vivo* showed that p-AKT expression decreased to 72.0 ± 3.1% of control levels in the CUR+LGG group, accompanied by increased FXR expression. Unlike conventional approaches that directly target signaling pathways using specific inhibitors, the present study modulates PI3K/AKT activity through a microbiota–metabolism regulatory mechanism. These findings support the existence of a “microbiota–bile acid–PI3K/AKT” regulatory axis, suggesting that CUR combined with probiotics may exert anti-resistance effects through this pathway. The combined intervention may promote a probiotic-dominated intestinal environment that limits the generation and accumulation of tumor-promoting secondary bile acids, thereby reducing endogenous exposure to these metabolites. This metabolic shift is associated with enhanced FXR signaling and suppression of PI3K/AKT activation. Future studies should apply metagenomic approaches to identify microbial species harboring bile acid metabolism–related gene clusters. In addition, FMT or colonization with defined bacterial strains in germ-free or antibiotic-treated animal models will be necessary to determine whether specific microbial taxa are sufficient to reproduce the observed metabolic changes and antitumor effects. Such experiments will help establish a direct causal relationship between microbiota alterations and bile acid–mediated signaling at the strain level.

The present findings highlight the central role of the PI3K/AKT pathway in mediating the reversal of chemoresistance. The combined intervention simultaneously suppressed AKT phosphorylation and reduced the expression of downstream effectors, including mTOR and GSK-3β. Such multi-level regulation may prevent resistance recurrence more effectively than single-target PI3K inhibitors by limiting compensatory pathway activation. Notably, our results suggest that LGG may indirectly regulate AKT signaling through microbial metabolites such as SCFAs, providing new insight into probiotic-mediated antitumor mechanisms. Compared with conventional AKT inhibitors [49, 50], the CUR–probiotic strategy may offer a safer and more physiologically integrated approach to pathway modulation while maintaining therapeutic efficacy. Nevertheless, the relative contributions of CUR and LGG to pathway inhibition remain to be quantitatively defined, and the potential tissue specificity of this regulatory mechanism requires further investigation.

Overall, this study demonstrates the synergistic antitumor activity of CUR combined with the probiotic LGG in a GEM-resistant GBC model. The combined intervention was associated with remodeling of specific gut microbial taxa, alterations in bile acid metabolism, and suppression of PI3K/AKT signaling. Bioinformatic prediction together with pharmacological validation revealed potential mechanistic links among these processes, highlighting FXR signaling as a key mediator connecting metabolic changes to downstream pathway inhibition. These findings provide experimental support for the involvement of a “microbiota–bile acid–PI3K/AKT” regulatory axis in the modulation of chemotherapy resistance in GBC. Consistent results from both *in vitro* and *in vivo* experiments suggest that the CUR–probiotic strategy exerts multi-dimensional regulatory effects on tumor progression and drug-resistant phenotypes.

LGG may enhance the systemic exposure of CUR through several potential mechanisms, including stabilization of the intestinal environment, modulation of drug transporters, inhibition of first-pass metabolism, and microbiota-mediated metabolic regulation. These effects likely increase both the amount and persistence of CUR in systemic circulation, thereby providing a pharmacokinetic basis for the enhanced biological activity observed with the combined intervention. After systemic absorption, CUR acts together with the microecological regulatory effects of LGG in the intestine to influence tumor progression through multiple pathways, particularly the “microbiota–bile acid–PI3K/AKT” regulatory axis, ultimately contributing to the reversal of chemoresistance. In this process, LGG appears to play dual roles. First, it may function as a bioenhancer, increasing the systemic exposure of CUR and facilitating sufficient drug delivery to tumor tissues. Second, LGG acts as a microecological modulator, reshaping the intestinal environment of the tumor host and influencing key metabolic and signaling pathways. The synergy between these two mechanisms provides a plausible explanation for why the therapeutic efficacy of the CUR+LGG combination exceeds the simple additive effects of the two interventions.

Beyond identifying PI3K/AKT as a potential molecular target of CUR through bioinformatics prediction, this study introduces an ecological perspective in which probiotics cooperate with natural compounds to remodel the gut microenvironment and enhance anticancer efficacy. These findings provide a conceptual framework for developing microbiota-based nutritional intervention strategies. From a clinical standpoint, the CUR–probiotic approach may represent a promising adjuvant therapy for GBC, particularly for patients with chemotherapy-resistant disease, given its favorable safety profile and potential translational applicability.

We acknowledge the well-recognized limitation of CUR regarding its poor oral bioavailability [51]. The *in vitro* concentration (40 μg/mL) and *in vivo* dose (500 mg/kg) used in this study were determined based on preliminary experiments and supported by extensive preclinical literature demonstrating antitumor efficacy and mechanistic relevance [52, 53]. Nevertheless, the plasma concentrations achieved under these conditions likely exceed those attainable through conventional oral administration in humans [54], limiting direct clinical extrapolation. Future studies should focus on improving CUR bioavailability through optimized delivery strategies, including co-administration with bioenhancers or the development of nanocarrier-based formulations, to achieve therapeutic efficacy at clinically feasible doses.

This study has several limitations. First, although bioinformatic prediction together with *in vitro* and *in vivo* validation suggested that the CUR+LGG combination reverses GEM resistance through suppression of the PI3K/AKT/mTOR pathway, the necessity and sufficiency of this pathway were not pharmacologically confirmed. Future work should combine CUR/LGG with PI3K or AKT inhibitors to evaluate potential synergistic or additive effects, and perform rescue experiments using AKT activators or pathway agonists to determine whether reactivation of the pathway reverses CUR/LGG-induced apoptosis and growth inhibition. These experiments would provide more direct causal evidence supporting the proposed “microbiota–bile acid–PI3K/AKT” regulatory axis. Second, the present work focused primarily on CUR. Other turmeric-derived analogs, including DMC and BDMC, may exhibit comparable or stronger chemosensitizing activity. Systematic structure–activity relationship (SAR) analyses will therefore be necessary to determine whether these derivatives possess improved therapeutic potential. Third, the potential influence of LGG on GEM pharmacokinetics or tissue distribution was not investigated. Future studies should quantify GEM and its metabolites to clarify whether microbiota-mediated mechanisms affect drug disposition and contribute to the observed reversal of chemoresistance.

It is noteworthy that, in addition to modulating gut microbiota composition and microbial metabolites to influence chemoresistance in GBC, both CUR and probiotics have been reported to possess immunomodulatory potential. Previous studies indicate that these agents can promote effector T-cell activation, suppress immunosuppressive cell populations, and regulate inflammatory cytokine production [55-58]. However, the present study primarily focused on elucidating a novel mechanistic axis—the gut microbiota–bile acid metabolism–PI3K/AKT signaling pathway—and systematically confirmed through *in vitro* and *in vivo* experiments that the CUR–probiotic combination effectively reverses GEM resistance. Consequently, comprehensive characterization of the tumor immune microenvironment was not performed, which represents a limitation of this study. Future investigations should incorporate immunological analyses, including profiling of tumor-infiltrating immune cells, cytokine quantification, and flow cytometric assessment of immune cell activity, to determine whether the CUR–probiotic strategy also modulates tumor immunity. These multidimensional approaches integrating microbiota, metabolism, signaling pathways, and immune regulation will provide a more comprehensive understanding of the underlying therapeutic mechanisms. Although correlation analyses and pharmacological modulation of FXR support the existence of a “microbiota–bile acid–FXR–PI3K/AKT” signaling axis, the strict causal relationships among these components remain to be fully established. For example, it remains unclear whether microbiota alterations exert their effects primarily through bile acid metabolism or whether PI3K/AKT inhibition represents the dominant downstream mechanism responsible for reversing chemoresistance. Addressing these questions will require more refined experimental strategies, such as colonization with defined microbial communities, cell-specific knockout of bile acid receptors, and multi-omics time-series analyses. In addition, a design consideration of this study should be clarified. *In vitro* experiments used LGG cell-free supernatant (CFS) to simulate the effects of its metabolic products, whereas *in vivo* experiments employed live LGG to evaluate its integrated effects within a whole organism. These two approaches reflect different mechanisms of action (direct metabolite effects vs. microecological remodeling driven by live bacterial colonization). Therefore, the *in vitro* results cannot be directly equated with the full effects of live bacteria *in vivo*, and vice versa. Although the data collectively support the effectiveness of the combined strategy, the study did not directly compare the relative contributions of live bacteria and CFS *in vivo*, which represents a limitation. Future studies should incorporate such comparisons to better clarify the mechanisms underlying probiotic action.

Finally, the present work did not systematically investigate dose-dependent effects of the CUR–probiotic combination or its applicability across different molecular subtypes of GBC. Future research should integrate multi-omics analyses and progress toward preclinical and clinical evaluation to facilitate the development of precision microecological intervention strategies.

## Supplemental Materials

Supplementary data for this paper are available on-line only at http://jmb.or.kr.
