# Supplementary material for: Microbiome Modulation with Lactobacillus rhamnosus GG Potentiates Curcumin's Efficacy in Reversing Gemcitabine Resistance of Gallbladder Cancer through Gut Microbiota-PI3K/AKT Axis
Source: J Microbiol Biotechnol. 2026 Apr 21;36:e2601007. doi: 10.4014/jmb.2601.01007 (PMC13102627; doi:10.4014/jmb.2601.01007)
Supplement: Supplementary file 1 [file jmb-36-e2601007-supple.pdf]

J. Microbiol. Biotechnol. 2026. 36: e2601007
https://doi.org/10.4014/jmb.2601.01007

Supplementary Figures

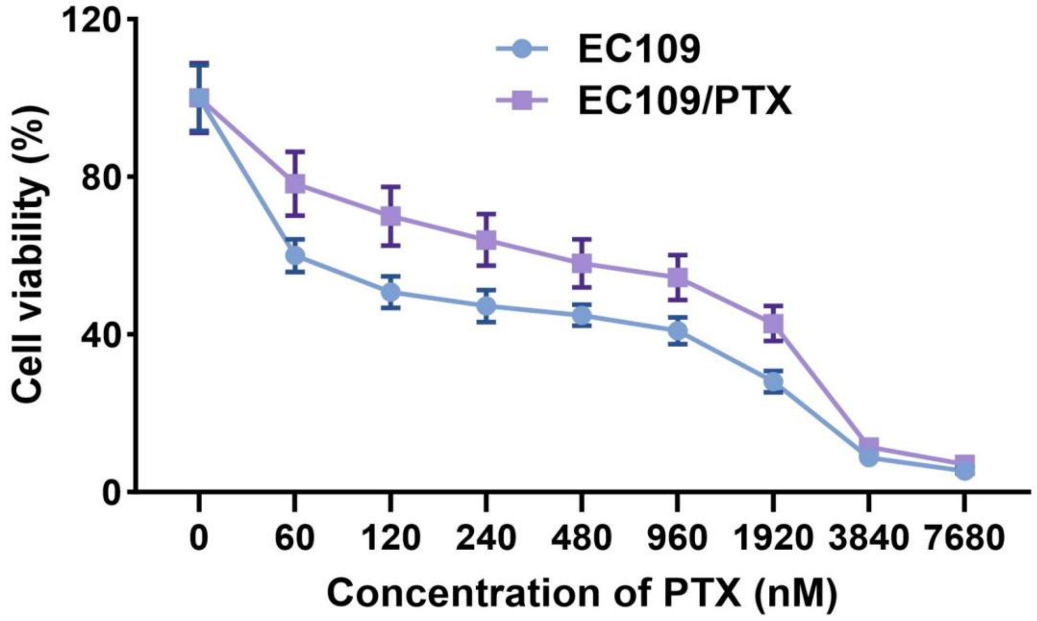

Fig. S1. Construction and validation of GEM-resistant GBC-SD cells.

Note: CCK-8 assay was used to evaluate the sensitivity of GBC-SD cells and GEM-resistant sublines to gemcitabine. Data are presented as mean ± standard deviation (Mean ± SD).

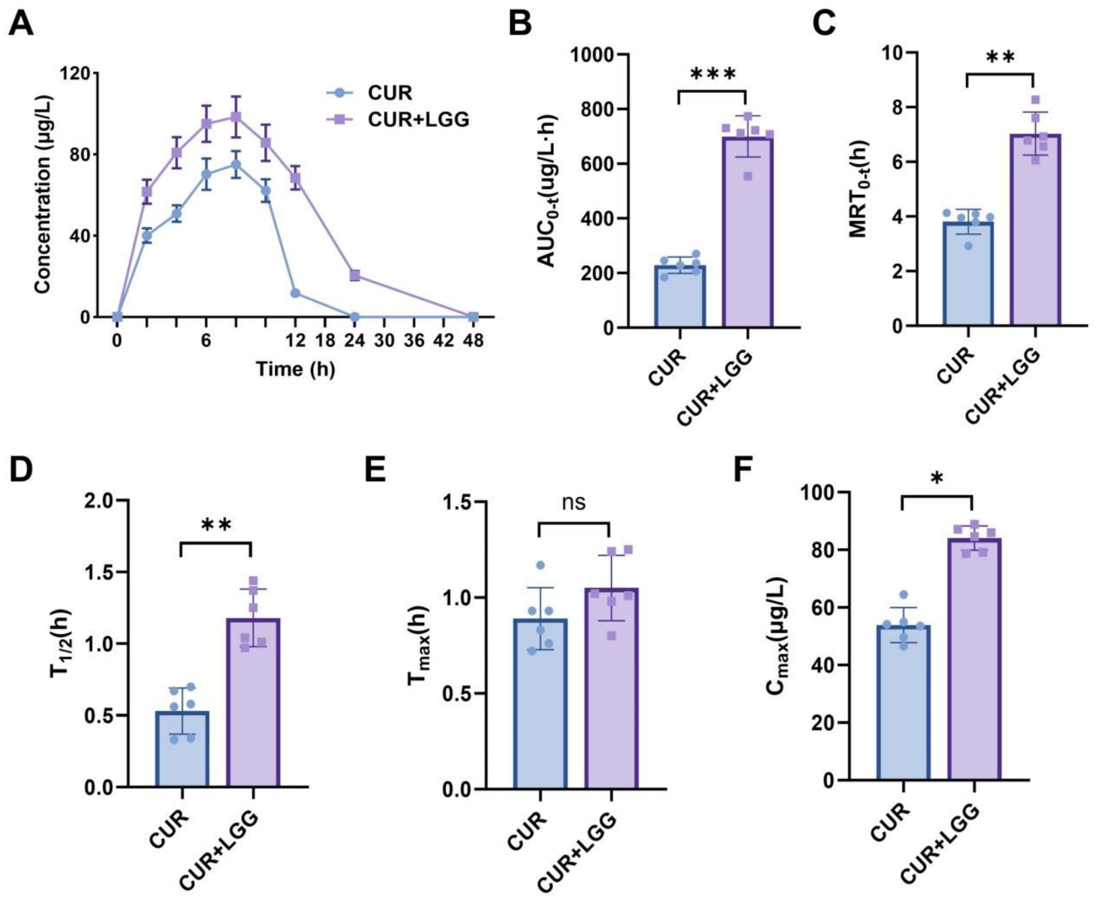

Fig. S2. Effect of LGG on the Pharmacokinetic Profile of Curcumin.

Note: (A) Mean plasma concentration–time curves of curcumin after oral administration in the two groups of mice (n = 6, mean ± SD); (B-F) Statistical comparisons of major PK parameters (AUC_0–t, MRT_0–t, $T_{1/2}$, Tmax, Cmax). *p < 0.05, **$p < 0.01$.

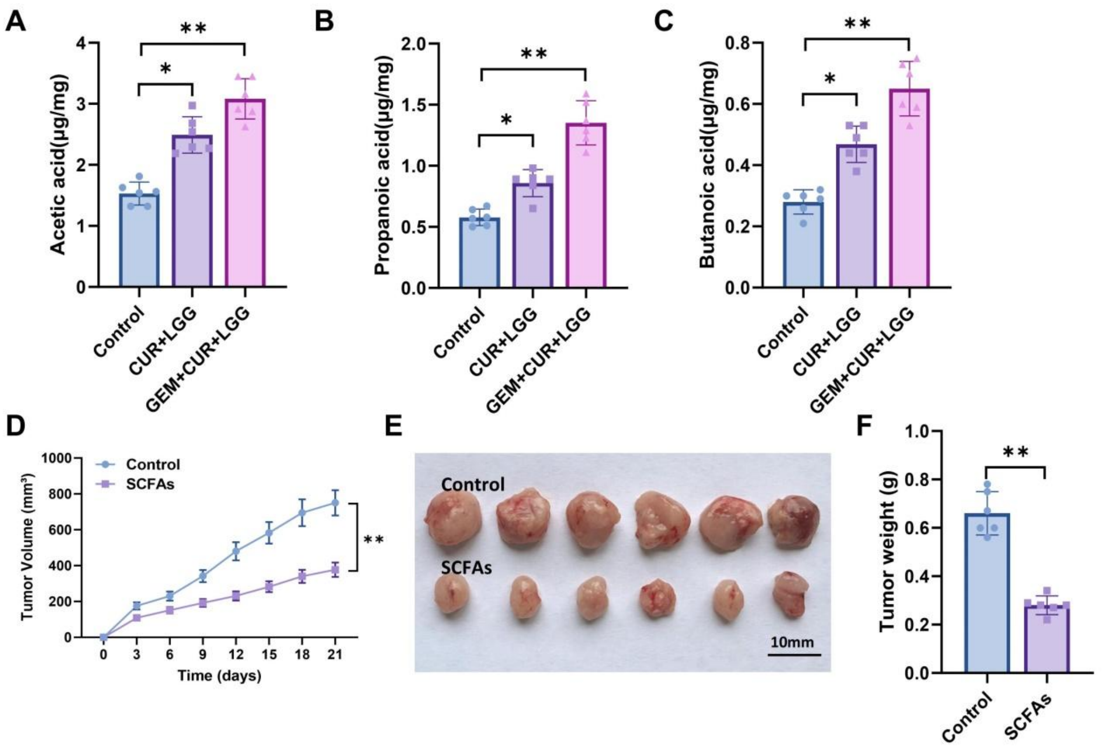

Fig. S3. Validation of the Role of SCFAs in the CUR+LGG Combination Intervention.

Note: (A-C) Fecal concentrations of SCFAs (acetate, propionate, and butyrate); (D–F) Effects of SCFA supplementation on tumor volume (D–E) and tumor weight (F). n = 6. Data are presented as mean ± standard deviation (Mean ± SD). *$p < 0.05$, **$p < 0.01$, ***$p < 0.001$.

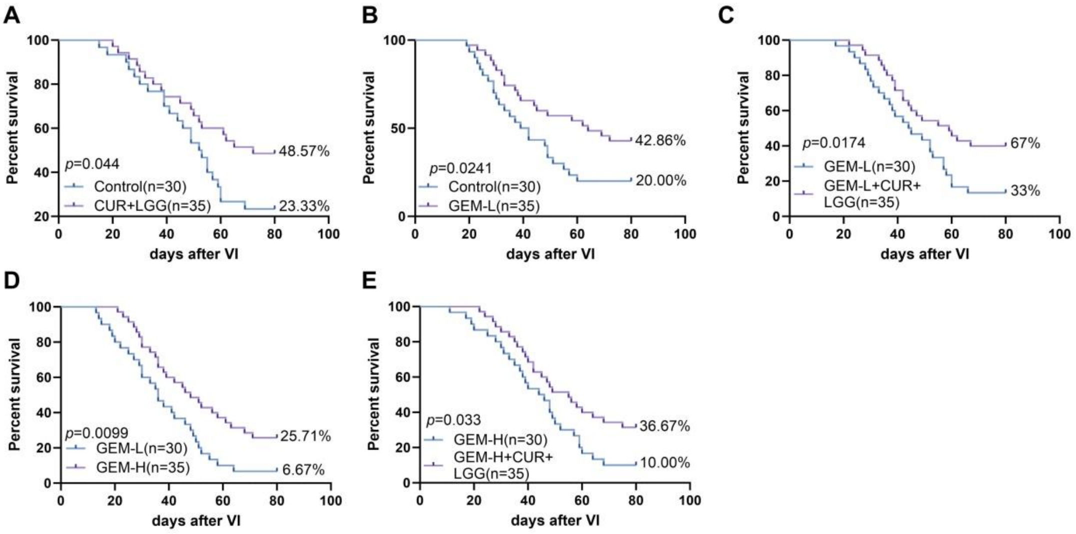

Fig. S4. Effects of Different Treatments on the Survival of GEM-Resistant Tumor-Bearing Mice.

Note: (A-E) Kaplan–Meier survival curves showing the survival rates of mice in each group. GEM-L: gemcitabine 25 mg/kg; GEM-H: gemcitabine 50 mg/kg. Survival curves were compared using the log-rank test. *$p < 0.05$, **$p < 0.01$.

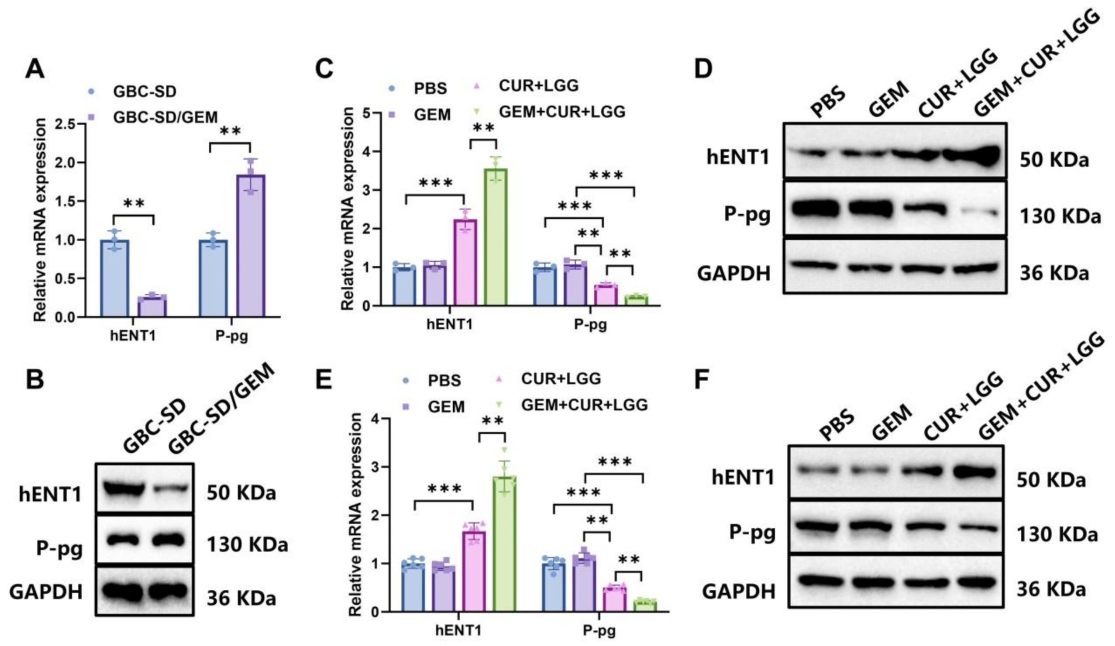

Figure S5. Regulatory Effects of CUR+LGG on the Expression of GEM-Resistance-Related Factors hENT1 and P-gp.

Note: (A-B) mRNA (A) and protein (B) expression levels of hENT1 and P-gp in parental and GEM-resistant cells; (C-D) Changes in mRNA (C) and protein (D) expression of hENT1 and P-gp in GEM-resistant cells *in vitro* under different treatments; (E-F) Changes in mRNA (E) and protein (F) expression of hENT1 and P-gp in tumor tissues from GEM-resistant tumor-bearing mice in vivo. The number of mice in each group was six (n = 6), and the cell experiments were performed in triplicate (n = 3). Data are presented as mean ± standard deviation (Mean ± SD). *$p < 0.05$, **$p < 0.01$, ***$p < 0.001$, ****$p < 0.0001$.
